# The Effects of Community Attachment and Information Seeking on Displaced Disaster Victims’ Decision Making

**DOI:** 10.1371/journal.pone.0151928

**Published:** 2016-03-23

**Authors:** Kong Joo Shin, Ryo Nakakido, Shinya Horie, Shunsuke Managi

**Affiliations:** 1 Kyushu University, Department of Urban and Environmental Engineering, School of Engineering, Fukuoka, Japan; 2 Tohoku University, Graduate School of Environmental Studies, Sendai, Japan; 3 Kobe University, Department of Economics, Kobe, Japan; University of Vermont, UNITED STATES

## Abstract

This paper uses original survey data of the Great East Japan earthquake disaster victims to examine their decision to apply for the temporary housing as well as the timing of application. We assess the effects of victims’ attachment to their locality as well as variation in victims’ information seeking behavior. We additionally consider various factors such as income, age, employment and family structure that are generally considered to affect the decision to choose temporary housing as victims’ solution for their displacement. Empirical results indicate that, ceteris paribus, as the degree of attachment increases, victims are more likely to apply for the temporary housing but attachment does not affect the timing of application. On the other hand, the victims who actively seek information and are able to collect higher quality information are less likely to apply for the temporary housing and if they do apply then they apply relatively later.

## Introduction

A large-scale natural disaster is physically destructive, and the recovery process is emotionally and financially costly. Survivors need to transition from victims to citizens, and decision makers are often held accountable for a speedy recovery [1]. Therefore, understanding the factors that promote effective disaster recovery is critical for both parties [2]. In particular, the displacement of disaster victims is an immediate and distressing concern for both victims and the government. Because emergency shelter is not a long-term solution and permanent relocation is not an option for many disaster victims, temporary housing is an important intermediate option between emergency shelters and permanent accommodations. Thus, the construction and distribution of temporary housing constitutes a vital component of the recovery process.

The provision of temporary public housing has become a standard procedure to resolve the displacement caused by disasters. Public discussion focuses on the government’s reaction to the damage, including whether the government has allocated sufficient resources to build and manage temporary housing. The media covers the quality of temporary housing, the costs paid by residents for it, and the timing of the discontinuation of temporary housing, but it rarely covers the decision making of victims to actually choose temporary housing as a displacement solution when other options are available. Previous studies have identified the effective factors that cause discrepancies in the overall progress of recovery at the individual and regional levels, and the implications are important to efforts to improve the recovery process. However, understanding the details of how survivors react to address displacement can shed some light to how decision makers should proceed when they face particular stages of the recovery process.

We contribute to the literature on disaster response and management by disaggregating the effects of various components of victims’ decisions to move into temporary housing as well as the timing of victims’ application for temporary housing. Our main objective is to empirically analyze the effectiveness and measure the impacts of the locality attachment and information-seeking behavior of disaster victims on the decision to apply for temporary housing and the timing of the application while controlling for the effects of other generally emphasized factors such as age, income and family circumstances. For our analysis, we use original survey data from the Great East Japan Earthquake disaster victims that were collected in March 2012, approximately one year after the earthquake.

The paper is structured as follows: Section 2 provides the background on disaster displacement and temporary housing, with a particular focus on the case of the Great East Japan Earthquake. Section 3 discusses the related literature and the expected effects of locality attachment and informational factors regarding disaster victims’ choice of a solution for displacement and the timing of their decisions. Section 4 and Section 5 provide details on the data and the statistical analyses, respectively. Section 6 discusses the results of the empirical analysis, and Section 7 concludes the study.

## Disaster and Displacement

One of the consequences of a natural disaster is the displacement of victims. The earthquake and tsunami that occurred in Japan on March 11, 2011 was the largest earthquake ever recorded in Japan. Registering a maximum seismic intensity of 7 and a moment magnitude of 9.0, the earthquake triggered a powerful *tsunami* with wave heights of up to 9.3 meters that inflicted enormous damage, particularly in the *Tohoku* region’s coastal area. According to official reported statistics, the earthquake resulted in 15,884 deaths, 2,633 missing persons and 6,148 injured victims. Furthermore, 127,302 structures collapsed and 272,849 structures were left in semi-collapsed conditions. The estimated total damage was 16.9 trillion yen (approximately 169 billion dollars), of which 10.4 trillion yen (approximately 104 billion dollars) of financial damage resulted from the destruction of physical property, including both residential and non-residential buildings and other land property damage.

The disaster led to the displacement of 470,000 people who were forced to stay in emergency shelters. The number of people in emergency shelters rapidly decreased over the six months following the disaster, at which time only 27,000 victims remained in the shelters, as the majority of the disaster victims had either found housing on their own or had moved to temporary housing. One year after the earthquake, approximately 320,000 disaster victims remained in temporary housing, and approximately 240,000 victims remained without permanent housing as of March 2014, three years after the earthquake.

The first supplementary budget for the 2011 fiscal year allocated approximately 4 trillion yen (approximately 40 billion dollars) to the recovery process. Of the total budget, approximately 362.6 billion yen (approximately 3.6 billion dollars) was earmarked for the construction of temporary housing. The initial plan was to offer approximately 100,000 units of temporary housing, more than double what was allocated after the *Hanshin-Awaji* earthquake. In the *Hanshin-Awaji* earthquake of 1995, 48,300 units of temporary housing were built over a seven-month period to provide housing for 100,000 out of an estimated 316,000 homeless refugees. The disaster victims were allowed to remain in the temporary housing for three years after the earthquake. However, after three years, 45 percent of the temporary houses remained occupied due to inadequacies in the supply of affordable permanent housing [3].

Although temporary housing is a common solution for displacement caused by disasters, it is not a permanent solution. During the emergency sheltering phase, living expenses are almost fully covered by outsiders, mostly by the government. During the temporary housing phase, people return to their normal work, school and daily activities while hoping for a more permanent solution [4]. Thus, temporary housing does not completely resolve the future uncertainty of disaster victims.

With no constraints, the optimal solution for disaster victims would be to immediately provide permanent housing. The reality, however, is that permanent housing is a costly option that frequently requires either long-term relocation or a long wait until the disaster sites recover and new houses are built. Depending on the circumstances and the preferences of disaster victims, their chosen solution for displacement varies; some choose to move into temporary housing, and some do not.

One of the most widely emphasized decision factors is variations in income. Temporary housing is heavily subsidized and thus less costly for victims than other options, particularly over the short term. The housing options for low-income individuals are restricted, whereas high-income individuals are more capable of finding new accommodations and resolving their displacement more quickly because they can afford more costly alternatives. The employment factor, which is closely related to income, also affects the choice of a solution for displacement due to disaster. Unemployed individuals, part-time workers and elderly people who are dependent on social security are more likely to be financially restricted. In addition, individuals in some jobs, such as public servants and individuals in agricultural jobs, are less mobile because of the location-specificity of their jobs. Therefore, disaster victims with jobs that are closely connected to a specific location and community remain in the locality and enter temporary housing until the locality recovers from the disaster.

The choice of a solution for displacement may be partially affected by the restrictions and the preferential treatment that occur in the application process for temporary housing. For example, the disaster victims in the *Hanshin-Awaji* earthquake had to live in an emergency shelter to qualify to apply for the temporary housing lottery. Moreover, to qualify for permanent government rehousing, a family had to be living in temporary housing at the time of application. Such restrictions may have created an intricate incentive structure for disaster victims when they were selecting a solution for their displacement. With respect to the Great East Japan Earthquake, the restrictions on the applicants were modified from previous disasters. Qualification was mainly based on the degree of property damage, and preferential allocation was based on age and the number of dependents. Hence, the age and family structure of potential applicants affect their decisions regarding whether to move into temporary housing.

## Solving displacement: The effects of attachment and information

In addition to the generally emphasized decision factors such as age, income and family circumstances, our analysis focuses on the variations in local attachment and the information-seeking behavior of disaster victims as explanatory factors regarding the choice of a solution for displacement. In this section, we review the related literature and discuss the expected effects of these two factors separately.

### Locality attachment

Although the concept of locality attachment is widely discussed in various disciplines, particularly in the fields of sociology and psychology, empirical studies connecting disasters and attachment are scarce. According to the attachment literature, a sense of attachment affects human behavior through the positive recognition of a sense of belonging, and various attachment concepts are defined according to what people feel they belong to. Place attachment is defined as the sense of belonging to a particular location, such as a house or a specific area. For example, according to [5], the concept of place attachment is “a positive affective bond or association between individuals and their residential environment and “emotional involvement with places.” Similarly, [6] defines place attachment as “an individual cognitive or emotional connection to a particular setting or milieu.”

In addition to place attachment, the literature also emphasizes social attachments and specifically the interaction between place and social attachment, which leads to the concept of community attachment and a distinction between the effects of place and social attachment [7–13]. The empirical results of previous works suggest that the effects of social attachment are greater than the effects of physical attachment when the aggregate attachment of an individual is considered. [14] claims that community attachment is closely related to the development of local social relations. Similarly, [11] shows that social and physical place attachment can be distinguished and that the social components affect the degree of attachment more than the physical attachment. Furthermore, the authors show that the “physical and social dimensions of attachment vary across various definitions of space that vary in size, ranging from the house to the city scale,” and they further demonstrate that individuals show the strongest attachment to their homes and the city in which they live. [15] also considers the effects of social interaction on attachment and shows that social interaction with neighbors is one of the main behavioral elements of attachment to a locality. Moreover, [16] shows that the locality attachment of an individual increases as the number of close friends who live nearby increases.

Social interactions and connections are powerful tools of problem solving, particularly during unforeseen shocks such as large-scale natural disasters. [2, 17] finds that social capital and networks play a critical role in disaster recovery, and areas with greater ‘volunteerism’ recover more quickly. [18] and [1] provide empirical evidence from the case of the *Hanshin-Awaji* earthquake that social ties and the sense of attachment to a *locale* are strong predictors of life recovery. Volunteerism and social connections through community participation directly relate to the concept of social capital proposed by Aldrich through ‘bonding’ between people in the community and people ‘linking’ themselves with their locality [2]. We construct an attachment indicator that aggregates place attachment and the ways in which residents relate themselves with their community.

We use several proxies of attachment in this study based on various indicators used by previous studies to measure the degree of attachment. The duration of one’s stay in a specific location or locality appears to be widely used as an indicator of place attachment as well as social attachment. For example, the number of years in a residence was first used by [19] as a proxy of place attachment and has been continuously used by other researchers as a robust indicator. [20] has shown that the duration of residence has direct positive effects on individual-level local friendships, attachment to community, and participation in local social activities. Furthermore, [21] argues that recent immigrants generally exhibit lower levels of social attachment, primarily because of their shorter tenure in the community. We also use proxies such as participation in community events and whether disaster victims interacted with friends and neighbors to solve the specific problems of applying for temporary housing or utilizing other options. Given the results of [22] that disaster victims who regularly read local newspapers were more likely to return to their original locality after the Great East Japan Earthquake even when facing unresolved issues such as nuclear leakage, we also consider the local newspaper proxy to measure the degree of attachment.

We expect the empirical results to show that, ceteris paribus, an increase in attachment increases the likelihood of disaster victims applying for local temporary housing. We also expect that disaster victims with strong attachments will apply earlier to move into temporary housing. Disaster victims with strong attachments are more likely to remain in the locality even if the area is heavily damaged. For those who want to remain in their locality, temporary housing is a valuable option because the reconstruction of houses and facilities takes time. Moreover, such disaster victims would apply earlier because they would spend less time considering alternatives other than remaining in their locality.

### Information-seeking behavior and information quality

Following a disaster, victims typically face difficulties collecting information relevant to their physical security as well as to their short-term and long-term stability in terms of housing, employment and providing for dependents. Disaster victims must process massive amounts of information regarding disaster damage and the availability of support from various sources; in addition, they must also determine what and how much information they will take in and then actually apply the information to their decisions.

Previous studies that link disaster and information emphasize the Internet as an important information collection tool during a disaster. [23] notes that “we cannot yet call the Internet the ultimate answer to our emergency communication problems. It would be more accurate to speak of the Internet as a useful and instructive prototype for what may eventually become a coherent emergency information infrastructure” [24]. However, recent studies linking information and communication technology (ICT) with crises identify the convincing effects of ICT on information-seeking activities and the quality of collected information. First, the strength of computer-mediated communication serves as a means of sharing relevant information among local people during periods of crisis. During the *Hanshin-Awaji* earthquake, the Internet remained available, although satellite communication dishes were crippled. In response to the potentially overwhelming burden of heavy electronic traffic, mirror sites were established around the world. These sites enabled users to continue to receive valuable information such as press releases, lists of missing persons, reports on the extent of damage, and the effects of mitigation efforts [25].

Second, according to [26], traditional media disseminates disaster-related messages regularly; however, the coverage of broadcasters and media journalists is biased and heavily focused on“human interest and other headline-grabbing stories.” Subjects who were surveyed by the authors during the Southern California wildfires in 2007 expressed concern regarding the difficulty of obtaining useful local information from traditional broadcast sources. Therefore, disaster victims frequently utilize social connections and the resources at hand and turn to their own communities for support; thus, ICT such as the Internet have played an important role in victims’ information-seeking activities during disasters [26, 27]. Community-based interactions through ICT facilitate extended public actions and serve as a source of functional and emotional support [28]. In particular, local information provided through ICT by people with local knowledge can provide more accurate and detailed and thus more useful information than broadcasting by the general media [29]. Additionally, compared with traditional media, ICT is more effective in building community resources and can facilitate long-term social cohesion in geographic communities [26].

Because the Internet is a valuable source of information during and after a disaster, how victims utilize the Internet should be considered a factor that determines the information-seeking behavior of disaster victims and the quality of collected information. We incorporate the related measures of Internet access and Internet usage patterns among disaster victims in our study and expect that those who were able to access the Internet and web sources or online communities were able to obtain more accurate information faster than victims without access who did not use ICT as a tool to obtain information despite the availability of the Internet. Although the Internet is given special importance in the analyses, the previous literature does not deny the impact of traditional information sources following a disaster. Therefore, we also use regular subscriptions to national newspapers and weekly magazines that are considered sources of high-quality information. The list of magazines excludes publications that are categorized as gossip magazines or comic books.

Collecting information is costly and time-consuming, so those who actively seek information place high value on having more information regarding different options and the details about available choices to justify the cost. Moreover, variations in access and the ability to collect information may affect information-seeking behavior. People with easier access to information who are more capable of using the relevant tools and have good organizational skills accrue a comparatively lower cost of information seeking and are thus more likely to actively seek information.

We expect that individuals who actively seek and have the ability to collect high- quality information are less likely to apply for temporary housing than their counterparts because such individuals are more likely to know of and consider options other than temporary housing. Because such information is scattered and likely to be disseminated separately and often privately by provider organizations, obtaining useful details regarding outside options without additional information collection is difficult. If it is reasonable to assume that those with more information have more options with which to address displacement, then even if not all information seekers choose alternative options, the relative probability of choosing temporary housing as a solution would be lower for those who know of more options than for victims who are less likely to be aware of alternative options.

Furthermore, we expect that if individuals with more information decide to apply for temporary housing, their decisions tend to be made later because collecting and evaluating information takes time, and these individuals are more likely to decide that waiting would actually allow them to collect more useful information to optimize their decision. There are several reasons why waiting may be beneficial. For example, there are variations in the timing of the construction of temporary housing. In the case of the *Hanshin-Awaji* earthquake, temporary housing construction lasted from two weeks to seven months [30]. Furthermore, there is also some evidence of inconsistent construction quality in temporary housing. According to [31], there were variations in the quality of temporary housing built for victims of the Great East Japan Earthquake, including differences in barrier-free amenities, temperature control, and the quality of bathrooms, playgrounds, community spaces, support centers and group homes. According to the report on temporary housing in *Iwate* prefecture, even at the same temporary housing site, the units that were added later were better built because changes were made in response to complaints regarding the existing construction [32]. Hence, it is reasonable to believe that there is some value to waiting and collecting additional information regarding temporary housing.

## Data

To empirically analyze the effects of location attachment and information factors on the likelihood of disaster victims applying for temporary housing and the timing of the application in the event that victims choose to apply, we utilize original survey data collected from disaster victims displaced by the Great East Japan Earthquake. The survey was conducted in March 2012, approximately one year after the earthquake.

The data collection process satisfies the requirements of the guidelines provided by the Ministry of Education, Culture, Sports, Science and Technology in Japan (MEXT). We did not seek approval from the university ethics committee because this survey is not clinical research that is designed to verify the effectiveness of newly developed medical drugs or medical equipment, nor is it research that studies a medical illness that requires official approval. However, given the special circumstances of our target respondents, we have constructed our questionnaire and conducted our recruiting process carefully to avoid causing any physical or mental stress. All respondents were required to read statements regarding the content of this survey and had to consent before completing the survey. We have not received any post-survey complaints that it caused physical harm or emotional distress.

We used a paper-based field survey to collect the data from displaced disaster victims who were living in the *Fukushima, Miyagi, and Iwate* prefectures, which were severely affected by the earthquake, and we used Internet surveys to reach displaced victims who had moved from the disaster area. We received 1,111 responses; out of the 1,111 total observations, 497 respondents applied for temporary housing and 614 respondents did not.

### Variables

This survey comprises a broad set of questions, including personal characteristics, victims’ financial and family circumstances, and subjective opinions regarding the recovery process. For the details of the variable construction, see S1 Table. We use two dependent variables in the analysis: Apply dummy = 1 if an individual *i* applied for temporary housing. This dummy distinguishes neither the timing nor the frequency of application. We also use *Timing* as the other main dependent variable, which ranges from 0 to 5, where the coded number indicates the phase number of the victim’s first application to the temporary housing entry lottery. We use Timing = 0 if *i* did not apply for temporary housing. Then, from 1 through 4 indicate the *n*_*th*_ housing lottery for which *i* applied. 5 collapses the 5_*th*_ to 16_*th*_ lottery phases. We have chosen 5 as a cut off and have aggregated the later stage lotteries because there were few observations after the 5th round of lotteries.

The main independent variables are the proxies of attachment and information. We use several factors to measure the degree of local attachment: 1) the number of years that *i* spent in the same house, 2) the level of participation and communication in community activities, 3) whether *i* had a family member who was a fireman (a fireman is considered to be particularly attached to a specific locality), 4) whether *i* currently holds, or held at the time of the crisis, a profession that is considered location-specific, such as local public servants, teachers, farmers and fishermen, 5) the number of local neighbors with whom *i* had discussed temporary housing post-disaster, and 6) whether *i* subscribed to local newspapers at the time of the earthquake. These variables were expected to cover a range of attachment concepts, including the place, community and social attachments of the disaster victims.

We also use a set of variables to measure how actively the victims were engaging in information-seeking behaviors and their capacity to collect high-quality information: 1) whether *i* had access to the Internet, 2) the degree to which *i* utilized the Internet for information-seeking activities, 3) whether *i* had a subscription to a national-level newspaper, and 4) whether *i* regularly read weekly magazines that are considered to have generally high information quality.

Because we use related variables to measure the concepts of attachment and information-seeking, we use the exploratory factor analysis method (EFA) to aggregate related variables to create the indices of attachment and information. EFA calculates the smallest number of unobserved interpretable factors required to explain the correlations among a set of variables without assuming linear relations among the observed variables. We use the latent variables that were identified as good factor indicators of the observed variables. Employing the EFA in this manner, we attempt to reduce the effect of multi-collinearity among the proxies of attachment and information and also to construct indices that emphasize the concepts rather than the lists of variables.

**Fig 1:**
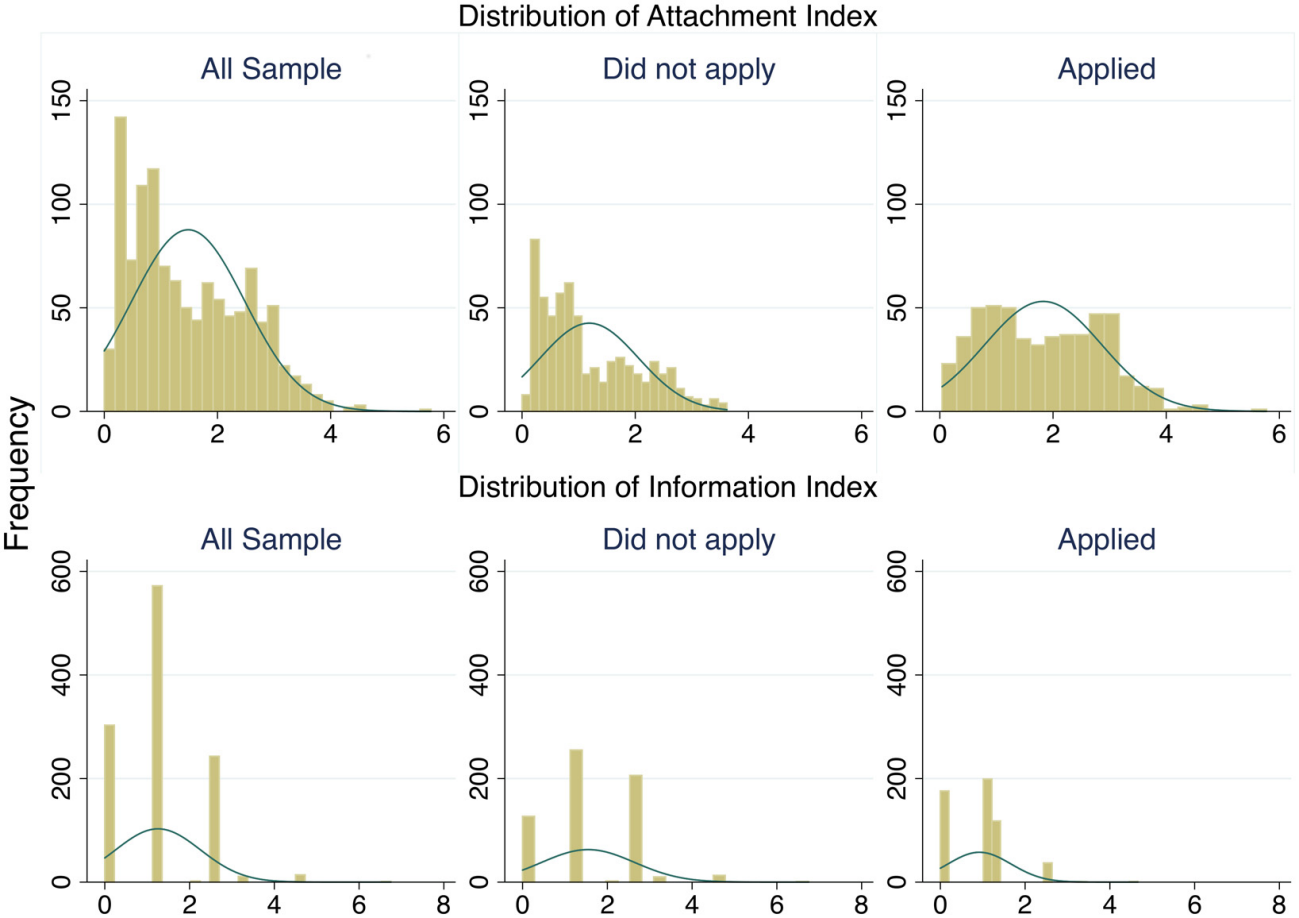
Distribution of attachment and information indices (normal-density plot).

Fig 1 shows the frequency distribution of the aggregated indices of attachment and information. Compared with the aggregated information index, the attachment index is more continuously distributed. This difference in dispersion patterns is expected because some attachment factors are continuous variables with relatively high variances; however, all of the proxies of information are either dummy variables or ordered variables with limited variation. The comparison of index distributions between those who applied and those who did not apply for temporary housing indicates that those with low attachment were more likely not to apply and that those who had applied had lower values on the information index.

As shown in Table 1, there are notable differences in the summary statistics between the group of victims who applied and the group of victims who did not apply. The mean values of the attachment factors, such as years in residence and the degree of community activity participation, are clearly higher for the sample of victims who applied for temporary housing, whereas the mean value of the information-seeking proxies, such as the dummy variable for regular national-level newspaper subscribers, indicates that those victims were clearly less likely to apply. A preliminary descriptive analysis appears to support our hypotheses of attachment and information effects on the likelihood of displaced victims choosing to apply for temporary housing: victims with high attachment and with low information-seeking behaviors were more likely to apply for temporary housing. The summary statistics for the whole sample are available in S2 Table.

**Table 1:** Summary statistics: by applied and did not apply.

	Did not Apply		Applied	
	mean	s.d.	mean	S.d.
Newspaper (local)	0.51	0.50	0.63	0.48
Years in residence	18.84	14.99	34.58	20.05
Community activities	0.36	0.65	0.73	0.85
Number of discussants	0.04	0.29	0.65	1.27
Fireman (dummy)	0.03	0.17	0.19	0.40
Local occupations	0.09	0.28	0.10	0.30
Newspaper (nation wide)	0.36	0.48	0.07	0.26
Weekly magazine	0.04	0.21	0.00	0.06
Internet access (dummy)	0.36	0.48	0.34	0.48
Internet information-seeking (dummy)	0.92	0.27	0.92	0.28
Internet information-seeking	2.55	0.67	2.56	0.67
Attachment index	1.19	0.87	1.83	1.03
Information index	1.55	1.10	0.92	0.75
Age	46.13	10.94	53.81	12.73
Family income	3.25	1.46	2.12	1.25
Number of family members	3.07	1.36	3.07	1.45
Income (per family member)	1.29	0.94	0.81	0.59
Number of dependents	1.72	0.96	1.77	1.27
Part-time employment	0.11	0.31	0.11	0.31
Retiree	0.04	0.20	0.15	0.36
Unemployed	0.05	0.23	0.21	0.41
Radiation	2.74	1.81	2.98	1.98
Observations	614		497	

The analysis also includes other control variables that may affect the likelihood of application for housing and the application’s timing. Past studies and media reports indicate that income and family structure have significant effects on decisions that are related to temporary housing and on general post-disaster relocation decisions. Thus, we control for the following individual characteristics: age, income (per family member), number of dependents, and occupation dummies (unemployed, retiree, part-time worker). In addition, given the large-scale nuclear reactor problem in *Fukushima* and the neighboring prefectures, we control for the subjective sensitivity of disaster victims regarding radiation to observe whether health concerns affected victims’ decisions to remain in the locality and apply for temporary housing. We also control for the prefectures’ fixed effects using dummy variables to control for other prefecture-specific but unobservable characteristics that affect the decision-making behavior of victims.

### Statistical Analyses

We use the logistic regression method to analyze decisions to apply and use the ordered logistic method as well as heteroskedastic ordered logistic methods to analyze the timing of applications. Table 2 shows the estimation results of the likelihood of disaster victims applying for temporary housing. Models (1) and (2) analyze the decision components of victims’ decisions to apply for temporary housing. Model (1) contains attachment and information-seeking-related variables, and model (2) includes aggregated indices of the attachment and information variables.

**Table 2:** Applying for temporary housing and the timing of application.

	(1) apply	(2) apply	(3) timing	(4) timing	(5) timing
Newspaper (nationwide)	-1.025*** (0.140)		1.432*** (0.308)		
Weekly magazine	-0.147 (0.526)		-0.050 (0.684)		
Internet access	-0.332*** (0.113)		-0.212 (0.301)		
Internet information seeking	-0.225 (0.187)		-0.583 (0.386)		
Information index		-0.507*** (0.066)		0.586*** (0.166)	0.902*** (0.197)
Years in residence	0.014*** (0.003)		0.000 (0.008)		
Newspaper (local)	-0.122 (0.102)		0.343 (0.275)		
Community activities	0.133** (0.067)		0.282* (0.163)		
Number of discussants	0.851*** (0.127)		-0.079 (0.100)		
Local occupations	0.019 (0.179)		0.270 (0.500)		
Fireman (dummy)	1.171*** (0.199)		-0.020 (0.345)		
Attachment index		0.325*** (0.052)		-0.041 (0.139)	-0.070 (0.140)
Income (per family member)	-0.448*** (0.124)	-0.382*** (0.106)	-0.164 (0.165)	-0.110 (0.198)	-0.144 (0.231)
Age	0.025*** (0.006)	0.027*** (0.005)	-0.047*** (0.014)	-0.043*** (0.013)	-0.040*** (0.015)
Number of dependents	0.045 (0.052)	0.042 (0.046)	-0.295** (0.132)	-0.286** (0.137)	-0.296** (0.144)
Part-time employment	0.352** (0.154)	0.189 (0.137)	0.066 (0.457)	0.004 (0.444)	-0.065 (0.442)
Retiree	0.643*** (0.195)	0.464** (0.182)	0.839* (0.461)	0.727* (0.435)	0.745* (0.450)
Unemployed	0.790*** (0.150)	0.611*** (0.145)	0.562 (0.357)	0.445 (0.333)	0.308 (0.348)
Radiation	-0.007 (0.031)	0.010 (0.028)	-0.069 (0.083)	-0.089 (0.081)	-0.329 (0.216)
lnsigma					
Information index					-0.236** (0.095)
Radiation					0.103* (0.057)
*N*	1111	1111	497	497	497

Standard errors in parentheses.

*p <.1,

**p <.05,

***p <.01

(1) & (2): logit regression, (3) & (4): ordered logit regression, (5): heteroskedastic logistic regression. All models include prefecture fixed effect.

Overall, the statistical significance of the variables varies little, and the direction of the effects remains consistent across the two models. Moreover, the signs are consistent with what we expect to observe; attachment positively affects the likelihood of application, and information-seeking negatively affects the likelihood of applying. Moreover, we find that several proxies of attachment have strong and robust effects on the decision to apply for temporary housing. Years in residence, which has commonly been used in previous studies as an attachment proxy, has a statistically significant positive effect. The number of respondents and the level of participation in community activities, which we use as a proxy for community network attachment, have a similar positive robust effect on the likelihood of applying for temporary housing.

Other location-specific occupations do not appear to have an effect on the decision to apply for temporary housing; however, the fireman dummy has a positive effect on the likelihood of applying for temporary housing. This result supports the general view that firemen have a strong occupation-specific local attachment.

Of the information-seeking variables, the most significant appears to be whether disaster victims read a national newspaper. Without differentiating types of newspapers, the coefficient on the newspaper dummy is statistically insignificant for newspapers; however, when we distinguish between national and local newspapers, a local newspaper has no effect and a national newspaper has an effect. These results imply that distinguishing the type of newspaper is important and that each type of proxy indicates separate concepts. In Japan, newspapers remain a common source of information. A regular subscription to a newspaper, particularly to a national newspaper, may proxy individuals’ attitudes, the degree of interest in information-seeking and the quality of obtained information. Furthermore, as discussed extensively in the previous section, Internet access appears to affect the likelihood of applying for temporary housing through information-seeking activities. Thus, the results appear to suggest that information-seeking and high-quality information reduce the likelihood of applying for temporary housing.

**Fig 2:**
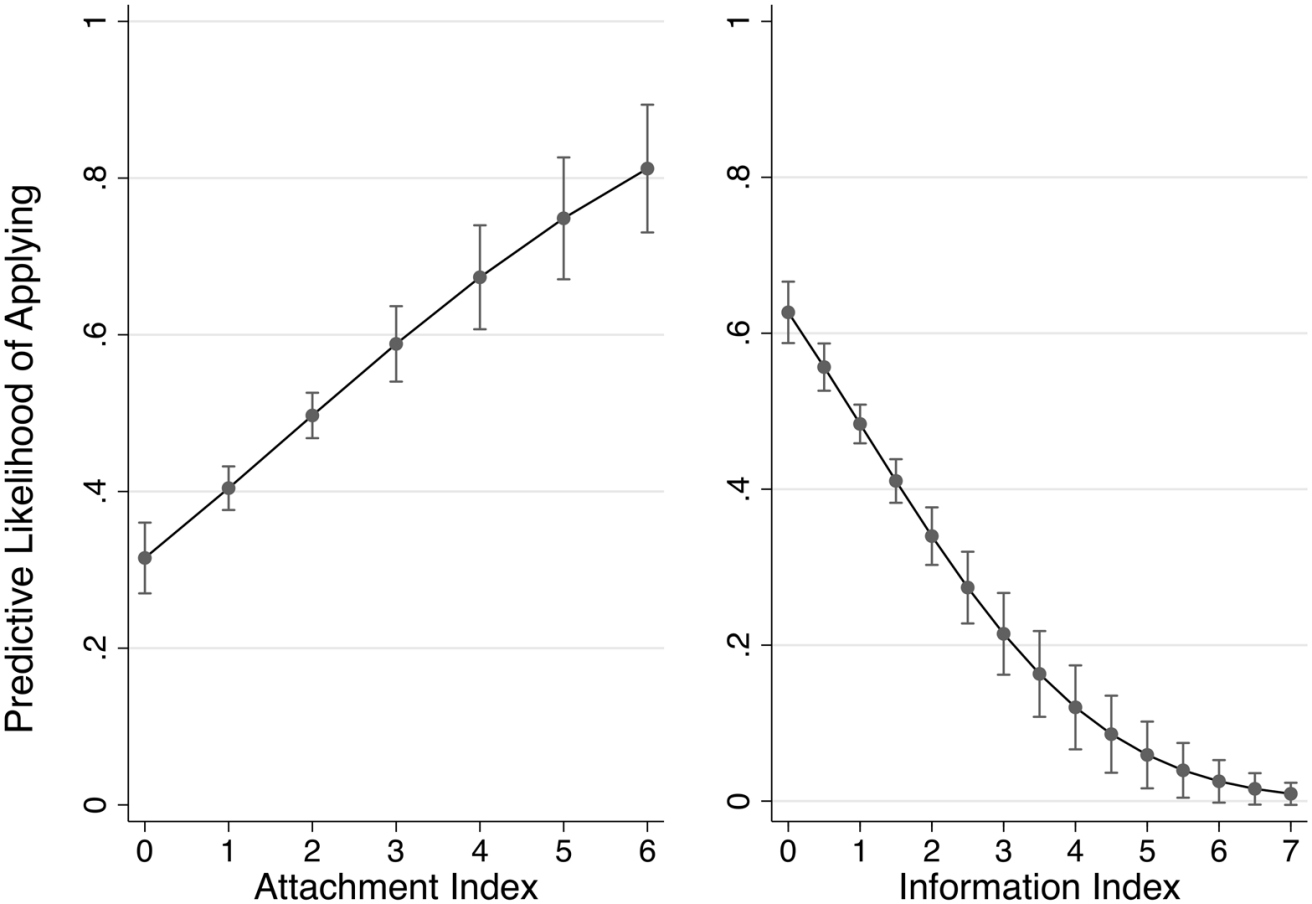
Predictive marginal effects of attachment and information indices. Predictive marginal effects and the likelihood of applying estimated from model (2) of Table 2 using factor polynomial fit. 95% CI is indicated for each unit of indices.

Fig 2 shows the marginal effects of the attachment and information indices on the likelihood of applying with a 95 percent confidence interval. The estimated marginal effects are calculated from model (2) of Table 2. Although both aggregate indices have highly significant effects on the decision to apply for temporary housing, there are clear opposite effects on the likelihood of applying: positive for attachment and negative for information seeking, with a relatively tight range of confidence intervals that indicates that the estimated effect is highly statistically significant and robust. The graph indicates that an individual with a relatively high degree of attachment is approximately 50 percent more likely to apply for temporary housing than a victim with minimal attachment and will nearly always decide to apply for temporary housing. Conversely, a victim with a high information index is approximately 60 percent less likely to apply for temporary housing than a victim with a low information index. Given the scale of the effects, the variations in both the attachment and information factors are important when we consider the behavior of displaced disaster victims regarding relocation. Additionally, we find that income level, age and other occupational variables affect the likelihood of applying for temporary housing. Low-income, elderly, and unemployed victims are more likely to apply for temporary housing.

We also analyzed the variations in application timing among victims who applied for temporary housing. Because *timing* is an ordinal variable and does not have equal interval intensity between the coded values, we use the ordered logistic regression method, which allows for adjustment for uneven differences between the values of ordinal variables.

Models (3) and (4) in Table 2 show the results of the ordered logistic analysis. The results of model (3) show the results related to the attachment and information variables; the only variable with more than a 95 percent statistical significance is the national newspaper dummy. This result implies that regular readers are more likely to apply for temporary housing during a later phase. Conversely, the indices show the clear effects of reading a national newspaper on the likelihood of applying for temporary housing. According to the results found in model (4), attachment does not affect the timing of the application, although an increase in the information index leads to a delay in the application timing.

The ordered logistic model assumes a proportional effect for each estimated cut level and does not consider the variation of level effects across explanatory variables. We can test whether the proportionality assumption, often referred to as parallel regression assumption, is violated using the Brant test. Table 3 presents the results of the Brant test. The results indicate that the information index and subjective radiation variable violate the proportionality assumption.

**Table 3:** Brant test of parallel regression assumption.

Variables	Chi2	p > Chi2
Attachment index	5.67	0.129
Information index	11.47	0.009
Income (per family member)	-6.85	-999.00
Age	0.35	0.950
Number of dependents	1.94	0.586
Part-time employment	3.25	0.354
Retiree	5.01	0.171
Unemployed	5.42	0.144
Radiation	19.52	0.000
All	94.45	0.000
Degree of freedom	33	

Therefore, using the ordered logistic model leads to inaccurate estimation results. To correct this fault and increase the robustness of the estimation results, we use the heteroskedastic ordered logistic regression method, also known as the ordered generalized least moments method. The results of model (5), which incorporates the violation of the proportionality assumption, show that the information index remains highly statistically significant and has an inverse effect on application timing. Furthermore, comparing the coefficients of models (4) and (5) indicates that the regular ordered logistic method underestimates the coefficient value of the information effect.

Additionally, the heteroskedastic ordered logistic regression method estimates the *lnsigma* value of the variables that failed the Brant test. The statistically significant negative coefficient of the *lnsigma* of the information index indicates that variations in timing among individuals decrease as the information index increases. In other words, as the information index increases, individuals consistently delay their application. Thus, compared with those with a low degree of information, the behaviors of individuals with a high degree of information exhibit a more uniform pattern.

**Fig 3:**
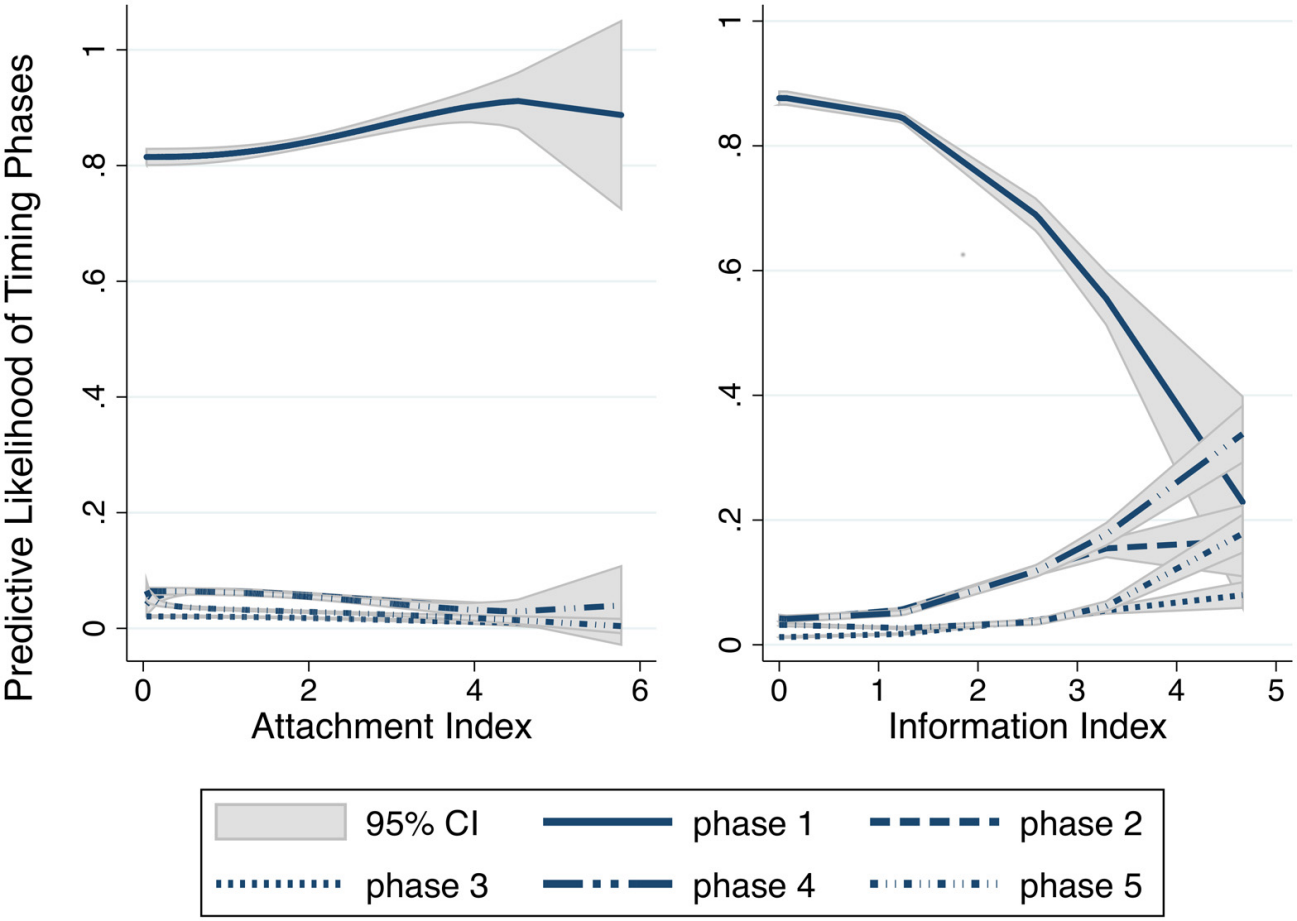
The marginal effects of attachment and information on the timing of application. Predicted Likelihood estimated from Model (5) of Table 2. Factor polynomial fit with 95% CI.

Fig 3 graphs the likelihoods of each timing phase for the ranges of the attachment and information indices using the results of model (5) from Table 2. The predictive likelihood of each phase remains quite constant across the range of attachment indices, thereby indicating that the change in the degree of attachment does not affect the timing of the application. However, a change in the information index affects the predictive likelihood of each phase. As the information index increases, 1) the likelihood of an individual applying during the first phase decreases dramatically, from approximately 90 percent to below 20 percent, and 2) the likelihood of applying during the later phases increases, particularly after the 4th round. The effect of a change in the information index is most apparent in the later phases, which is consistent with the implication of a negative *lnsigma* value.

## Result Discussion

Based on the results of previous studies, we expect to find the following: 1) as the degree of attachment increases, victims are more likely to apply for temporary housing and to apply earlier, and 2) as victims seek information more actively and are able to collect more and higher quality information, they are less likely to apply for temporary housing, and when such better informed victims do apply, they apply later.

The results of the analysis suggest that attachment matters in deciding whether to move into temporary housing but not with regard to the timing with which victims apply. This result deviates from the expected effects of attachment. One of the reasons why attachment does not affect timing is the relative dominance of other factors in the timing decision. The results show that the information factor has a significant effect on the timing decision. Moreover, although the income effect disappears when we consider variations in application timing, the number of dependents and age emerge as main decision factors in the timing of the application. This result is consistent with what the media has documented and emphasized regarding the hardships of families with children and elders in emergency shelters. Moreover, families with children and the elderly are likely to receive preferential treatment during the selection process for temporary housing. Therefore, victims without dependents may apply at a later stage with the understanding that they have a relatively slim chance of being selected as occupants; thus, they may decide to use more of their time to explore better outside options.

Unlike attachment, the information factor affects both the likelihood and the timing of applications, as expected. Victims who actively seek high-quality information are less likely to apply for temporary housing and are more likely to apply during later phases if they do apply. Victims who seek information are more likely to derive beneficial effects from doing so and are aware of more options. They also collect further information regarding their options and find value in waiting to obtain more information. Therefore, the solution for displacement that they choose is less likely to be temporary housing; moreover, if they do choose to apply for temporary housing, they tend to take longer to make that decision.

In addition, we find that highly informed victims, measured by the information index in the analysis, behave more uniformly than their counterparts in terms of timing. This result may be partially explained by the combination of delay factors that were previously discussed and the factors that induce victims not to apply too late. Victims generally stay in emergency shelters immediately following a disaster. In particular, we observe that the application timing of victims is concentrated in the 3rd and 4th application periods. Problems such as lack of privacy, inadequate heating and cooling facilities and inadequate living space make victims want to move out of emergency shelters as soon as possible [31]. The empirical results suggest that victims with a high information index are willing to skip the first and second stages of application and wait for the later phases; however, the incentives to move out of emergency shelters appear to increase following the early application periods.

## Conclusion

Understanding disaster victims’ behavior is important when considering the distribution policies of resources during the recovery process. To enhance our understanding, we used a unique individual survey dataset collected from displaced victims of the Great East Japan Earthquake. Variations in disaster victims’ behavior with regard to the decision to apply for temporary housing and in the timing of that application indicate that depending on their individual characteristics and circumstances, individuals value temporary housing differently as an option to resolve their displacement problems.

The results from the empirical analysis show that both attachment and information factors affect the victims applying for temporary public housing. In terms of application timing, contrary to the prediction, the attachment factor did not have an effect. Conversely, disaster victims with more information apply later. Moreover, we observe that the behaviors of victims with more information are more uniform and their application timing is concentrated in later phases compared with the scattered application timing of victims with less information.

From these results, we can draw several conclusions to consider in the disaster recovery process and in future studies. First, a high degree of local attachment increases the demand for temporary housing, depending on the damaged area. If a crisis occurs in a more newly developed area in which residents are likely to have low attachment, there will be comparatively less demand for temporary public housing with respect to the residents of that particular location. Second, the results of the statistical analysis indicate that the timing with which demand is expressed is determined by the family-related circumstances and the information quantity and quality that victims can access rather than the degree of attachment. Therefore, knowledge regarding variations in the information valuation of disaster victims would help governments and other relief organizations that assist with recovery processes to determine resource allocations during the recovery period.

We have focused on attachment and information factors, but there are additional factors that may also affect displaced victims’ decision behavior. [33] provides empirical evidence that recovery speed varies depending on the number of powerful politicians representing an area in the national government. If clientelism plays a role in the recovery process, it may cause a discrepancy in the availability of displacement solutions for the victims and may also affect the expectations of victims in terms of the quality of the options that may receive, which in turn will affect their decision making. Further accumulation of similar data regarding natural disaster displacement is also important, as it would help determine the optimal supply of temporary housing and the allocation of resources for disaster recovery as well as for providing disaster-related information.

## Supporting Information

S1 Table Variable List. Related survey questions and comments on coding.
(PDF)

S2 Table Summary Statistics. Summary statistics calculated using all sample.
(PDF)

S1 Data Raw Data. Data file (csv) includes all variables used in statistical analyses.
(CSV)

S1 Survey Questionnaire GEJE Displacement Survey Questionnaire. Questionnaire document in Japanese and English.
(PDF)

S1 Internet Survey GEJE Displacement Survey web html. Japanese only.
(PDF)
