# Supplementary material for: The Effects of Community Attachment and Information Seeking on Displaced Disaster Victims’ Decision Making
Source: PLoS One. 2016 Mar 23;11(3):e0151928. doi: 10.1371/journal.pone.0151928 (PMC4805184; doi:10.1371/journal.pone.0151928)
Supplement: S1 Table — Related survey questions and comments on coding. (PDF) [file pone.0151928.s001.pdf]

Table S1.Variable List. Related survey questions and comments on coding.

Variable	Related survey questions	Coding
Apply (dummy)	Have you ever applied for temporary housing before?	coded as 1 if yes, 0 if no.
Timing, Timing (dummy)	At which round did you apply for temporary housing? Please select all rounds in which you have applied	The options range from 1st to 16th rounds. We coded the earliest stage in which disaster victims have selected, then coded 1st-4th rounds as it is and we aggregated 5th round and up and coded as 5.
Newspaper (local, national), Weekly Magazine	Please let us know the name of publications that you read.	We categorized the publications into local, national newspaper and magazines. We coded weekly magazine dummy as 1 if the magazines that the victims regularly read are considered informational and high quality. We coded weekly comics magazine and the other magazines that are considered gossip magazines as 0.
Internet Access (dummy), Internet Information Seeking, Internet Information Seeking (dummy)	How much did you use Internet per day prior to the earthquake disaster?	4 options were available. 0: Didn't use Internet, 1: Just checking email , 2: Checking emails and a portal site (Yahoo!Japan, MSN Japan etc), 3:Checking emails and portal sites, and collecting information through net-surfing. We coded Internet access dummy as 1 if option 1 or 2 or 3 were selected and 0 otherwise. *Internet Information Seeking* is coded according to the intensity of Internet usage. with option number. Internet Information Seeking dummy is coded 1 if options 2 or 3 for Internet Information Seeking were selected and 0 otherwise.
Years in Residence	How long have you lived in the specific residence before you were displaced by the earthquake?	-
Community Activities	How often do you participate in local activities (example: local festivals or other organized events) other than local fire company activities?	3 options were available and coded accordingly: 0 if rarely participates, 1 if participates but does not communication with others especially the seniors in the community, 2 if participates actively and communicates with seniors in the community.
Fireman (dummy)	How many of your family members belong to a local fire company before the earthquake?	0 if none, 1 otherwise.

Variable	Related survey questions	Coding
Number of Discussants	Have you discussed regarding solutions to your displacement with non-family members? If you answered ‘yes’ to the previous question, please think of 5 people who you communicated about the solution for the displacement. How many of them currently live in temporary housing?	Coded as 0 if the victims did not discuss with non-family member, if victims discussed with someone then the answered number is coded.
Age	Please tell us your age.	-
Family Income	How much is your annual household income with tax (including pension)?	-
Number of FamilyMembers	How many family members are living together including you in your household?	-
Income (per family member)	-	Family income divided by the number of family members.
Number of Dependents	How many of your family members are 65 and older? How many of your family member is younger than 18 years and who attend educational institutions?	The numbers from both questions were added.
Part-time employment, Retiree, Unemployed, Local Occupations	Please select your occupation from the following options.	13 specific options were available and 14th option was to write-in. Local occupation is coded as 1 if following options were selected: agriculture and forestry (full-time), fishery(full-time), public servants, school teachers AND company worker / self-employed / government workers / school teachers who also work in agriculture and forestry or fishery. 0 otherwise.
Radiation	We would like to ask you about connection between awareness of radiation and your diet at home. Please select one of the following options that describes your answer the best.	Rating options from 1 to 8 were given where low score implies low awareness of radiation and high score implies high awareness. Score 1 is“doesn’t care about radiation” and score 8 is “worries so much about radiation that all of family members eat food that are surely unaffected by the radiation, which is supplied through special routes.”
Interview		Coded as 1 if directly interviewed at temporary housing sites and coded as 0 if interviewed by Internet survey
