# Supplementary material for: The Effects of Community Attachment and Information Seeking on Displaced Disaster Victims’ Decision Making
Source: PLoS One. 2016 Mar 23;11(3):e0151928. doi: 10.1371/journal.pone.0151928 (PMC4805184; doi:10.1371/journal.pone.0151928)
Supplement: S2 Table — Summary statistics calculated using all sample. (PDF) [file pone.0151928.s002.pdf]

Table S2. Summary Statistics. Summary statistics calculated using all sample.

	mean	s.d	min	max
Apply (dummy)	0.46	0.50	0.00	1.00
Timing	0.63	0.94	0.00	5.00
Newspaper (local)	0.56	0.50	0.00	1.00
Years in residence	26.13	19.19	0.00	81.00
Community Activities	0.53	0.77	0.00	2.00
Number of Discussants	0.32	0.94	0.00	5.00
Fireman (dummy)	0.11	0.31	0.00	1.00
Local Occupations	0.09	0.29	0.00	1.00
Newspaper (nation wide)	0.23	0.42	0.00	1.00
Weekly Magazine	0.03	0.16	0.00	2.00
Internet Access (dummy)	0.35	0.48	0.00	1.00
Internet Information Seeking (dummy)	0.92	0.27	0.00	1.00
Internet Information Seeking	2.55	0.67	0.00	3.00
Attachment Index	1.48	1.00	0.00	5.77
Information Index	1.26	1.00	0.00	6.75
Age	49.70	12.40	20.00	84.00
Family Income	2.73	1.48	1.00	8.00
Number of Family Members	3.07	1.40	1.00	7.00
Income (per family member)	1.07	0.84	0.14	8.00
Number of Dependents	1.75	1.11	0.00	6.00
Part-time employment	0.11	0.31	0.00	1.00
Retiree	0.09	0.29	0.00	1.00
Unemployed	0.12	0.33	0.00	1.00
Radiation	2.85	1.90	1.00	8.00
Interview	0.46	0.50	0.00	1.00
Observations	1111			
