# Supplementary material for: The Effects of Community Attachment and Information Seeking on Displaced Disaster Victims’ Decision Making
Source: PLoS One. 2016 Mar 23;11(3):e0151928. doi: 10.1371/journal.pone.0151928 (PMC4805184; doi:10.1371/journal.pone.0151928)
Supplement: S1 Survey Questionnaire — Questionnaire document in Japanese and English. (PDF) [file pone.0151928.s004.pdf]

Survey of the Great East Japan Earthquake and Displacement

2012.3

Contact Information:
Shinya Horie
Graduate School of Environmental Studies
Tohoku University
Email: horie4@gmail.com

S1あなたのお住まいの都道府県は。

Which prefecture do you currently reside? Please check one from the list of prefectures below.

____

S2 東日本大震災で、あなたの生活には以下のような影響がありましたか

The following is the possible effects of Tohoku earthquake on your lifestyle. Please mark the applicable ones to you.

☐	Experienced to live in an evacuation center.	避難所生活を経験した
☐	Moved my home to another place	自宅を移転した
☐	Experienced blackouts, which is not a part of planned rolling blackouts.	停電（計画停電でない）を経験した
☐	Experienced rolling blackouts	計画停電を経験した
☐	Experienced water outage	断水を経験した
☐	Experienced gas outage	ガスが使えない状態を経験した
☐	Could not go back home on the date when the earthquake happens, although finally reached home at the end	帰宅難民になった
☐	Stock up on water	水を買いだめした
☐	Stock up on rice	お米を買いだめした
☐	Bought new items to save electricity	節電のために何かを購入した
☐	Experienced something not listed above	上記以外のことを経験した
☐	Did not affected in particular	震災による影響は特になかった

Q1_1 What is your gender?

☐	Male	☐	Female

Q1_2 What is your age?

____________________ years old

Q2 あなたが現在お住まいの住宅は、震災前のあなたのお家と比べてどちらにあり

ますか。

Where do you live after Tohoku earthquake relative to the place you lived in?

☐	Same prefecture and same municipal	同じ県の同じ市内
☐	Same prefecture and different municipal	同じ県の市外
☐	Other prefecture	他県

Q2SQ1 あなたは過去に仮設住宅に応募された経験がありますか

Have you ever applied for the occupancy of prefabricated temporary housing provided by the government?

☐	Yes	☐	No

Q3 あなたが仮設住宅入居に応募したのはいつのことですか。現在仮設住宅にお住まいでない方は、仮設住宅入居に応募された経験がある場合お答えください。

When did you apply for the occupancy of a prefabricated temporary housing provided by the government? Even if you currently do not live in a prefabricated temporary housing but you have any experience of the application, please answer the date.

Year		Month		Day	

Q4 あなたは現在の住宅に入居する前に当選した仮設住宅への入居を辞退されたことはありますか。

Do you have any experience to decline the offered occupancy of a prefabricated temporary housing provided by the government?

☐	Yes	→ Go to Q5.
☐	No	→ Skip Q5.

Q5その理由は次のいずれですか

If you choose “Yes”, what is the reason leading you to decline the offer?

☐	The location of the offered a prefabricated temporary housing had similar characteristics of the house I used to live. For example, the offered prefabricated temporary housing is located near the sea.	当選した仮設住宅の位置が、被災した当時の家と似ていた（海に近いなど）
☐	The offered prefabricated temporary housing was located too far from my working place.	当選した仮設住宅が自分の職場から遠すぎた
☐	The location of the offered prefabricated temporary housing would make my family to get medical	当選した仮設住宅では、家族のための医療のケアが難し

	service	かった
☐	I had some concerns whether to be able to get along with amembers of a new community at a prefabricated temporary housing area.	当選した仮設住宅で、新しいコミュニティーに参加することに不安があった
☐	When the prefabricated temporary housing was offered, I was not financially ready to start the life without the support available at a evacuation center.	辞退した時点では、生活をはじめるための経済的な条件があなた自身にそろっていなかった（金銭面で生活を始める条件が揃っていなかった）
☐	When the prefabricated temporary housing was offered, there were not enough facilities supporting the life of my family surrounding of the housing. For example, there were not stores or any facilities providing items necessary to my family near the housing..	辞退した時点では、生活をはじめるための経済的な条件が周囲の環境にそろっていなかった（物を調達するための施設が近くに揃っていなかった）
☐	Other reason than the ones listed above. (Please write your reason.)<br>____	その他

Q6 あなたのご家庭の現在同居している方の人数は、あなたを含めて何人おられますか。

What is the number of your family members living with you including you?

☐ One ☐ Two ☐ Three ☐ Four

☐ Five ☐ Six ☐ More than seven

Q7あなたのご家族に、65歳以上の方は何人おられますか。

How many of your family members are older than 65?

☐	Zero	☐	One	☐	Two	☐	More than three

Q8 あなたのご家族に、18歳以下で教育機関に通われている方は何人おられますか。

How many of your family members are younger than 18 years old and going to school?

☐.	Zero	☐	One	☐	Two	☐	More than three

Q9 このうち、被扶養者（主に生計を支える人以外の人）は何人ですか

How many of your family members younger than 18 years and going to school are non-working dependents?

☐	One	☐	Two	☐	Three	☐	Four	☐	Five	☐	More than Six

Q10あなたのご職業は次のどちらですか

Employment status: What is your occupation?

☐	会社員	☐	農林業（専業）	☐	漁業（専業）	☐	自営業
☐	Corporate employee	☐	Agriculture or Forestry (fulltime)	☐	Fishery (fulltime)	☐	Self-employed
☐	公務員・教職員	☐	会社員と農林又は漁業との兼業	☐	自営業と農林又は漁業との兼業	☐	公務員･教職員と農林又は漁業との兼業
☐	Civil-servic e worker/teac her	☐	Getting income by working as a corporate employee and working as a farmer/fisherman/ forest labor at the same time	☐	Getting income by working as a self-employed worker and working as a farmer/fisherman/ forest labor at the same time	☐	Getting income by working as a Civil-service worker/teacher and working as a farmer/fisherman/ forest labor at the same time
☐	パート・アルバイト	☐	学生	☐	専業主婦	☐	年金生活
☐	Part-timer	☐ .	A student	☐ .	A homemaker	☐ .	Retired and receiving pension
☐ .	無職	☐ .	その他（具体的に）				
☐ .	Out of work	☐ .	Others (____________)				

Q11 あなたが震災以前に住んでおられた土地に、震災に遭われるまで何年お住まいでしたか。

How many years did you live in the place, where you lived before the Tohoku earthquake, by the earthquake occurs?

_____________Years

Q12 あなたが震災以前に住んでおられた土地には、ご親族のうちであなたが初めての代ですか。

Are you the first generation, who live the area where you lived before Tohoku earthquake, among all the generations of your families?

☐	Yes	☐	No

Q13 あなたが震災以前に住んでおられた土地には、あなたはご親族の代から合計で何年お住まいでしたか。

How many generations of your family including your generation do live in the area where you lived before Tohoku earthquake?

_____________Years

Q14 震災以前、あなたのご家族に消防団に所属している方は何人おられましたか

How many of your families did belong to a volunteer fire fighting team of your town before Tohoku earthquake happened?

____

Q15 あなたが消防団以外の地域の活動（祭やイベントなど）に参加する頻度はどの程度ですか。

How frequently do you participate in activities of a local community other than a volunteer fire fighting service?

☐	Participate in activities and have occasions to talk with members of an old generation.	積極的に参加していて、古参の人と話をする機会がある
☐	Participate in some activities and do not have occasion to talk with members of an old generation.	参加しているが、古参の人と話をするほどではない
☐	Rarely participate in any activities	あまり参加しない

Q16 あなたが仮設住宅入居に応募したとき、重視されたことを３つお答えください。

When you applied for the occupancy of a prefabricated temporary housing provided by the government, what did you consider the most important attributes of the prefabricated temporary housing? List up the most important three attributes for you.

1.	

2.	
3.	

Q17 東北地方太平洋沖地震（2011年3月11日に発生した地震）以外に、地震以外を含む（台風など）あなたの記憶に強く印象に残っている災害を5つお答えください。正確な名称である必要はありません。

There have been devastating disasters, of which kinds include earthquake, tsunami, typhoon, flood and so forth, in Japan. Please name 5 disasters other than Tohoku earthquake, which are the most memorable for you. Names of the disasters do not have to be correct.

Q18 Q17.でお答えになった災害のうち、実際に体験されたものはどれですか

Which disaster did you actually experienced among the disasters you named in Q17?

Q19 放射能汚染への意識と食事の関係についてご質問します。あなたのお家の食事では、次のうちどのような形態をとっておられますか。（ひとつだけ）

The following questions ask you about your perception about radioactive contamination of food and how you obtain food. Which of the following statements does apply to your perception and situation?

☐	I do not have any concern about radioactive contamination of food. I try to get locally produced food as much as possible.	放射能汚染は全く気にしていない。食材は可能な限り地産地消を意識して購入している。
☐	I have some concern about radioactive contamination of food. However I do not take any special efforts to explore new purchasing roots to get food other than locally produced food.	放射能汚染は少し気にしているが、特別なルートで食材を調達してはいない。
☐	I have some concern about radioactive contamination of food. However I do not have any special purchasing roots available to avoid locally produced food.	放射能汚染は少し気にしているが、特別なルートで食材を調達できない。
☐	I have some concern about radioactive contamination of food. With respect to some specific food, I try not to give locally produced one to children and women who are pregnant or women who want to get pregnant in the future. I have special purchasing roots for them.	放射能汚染は少し気にしている。特定の食材については、特別なルートで調達を行い、子供や妊娠を意識する女性については特別に調達した食材を用いている。
☐	I have some concern about radioactive contamination of food. With respect to some specific food, I try not to give locally produced one to any of my family members. I have special purchasing roots for them.	放射能汚染は少し気にしている。特定の食材については、特別なルートで調達を行い、家族全員同じものを食べている。
☐	I have a lot of concern about radioactive contamination of food. However I do not have any special purchasing roots available to avoid locally produced food.	放射能汚染を非常に気にしているが、特別なルートで食材を調達できない。
☐	I have a lot of some concern about radioactive contamination of food. With respect to some specific food, I try not to give locally produced one to children and women who are pregnant or women	放射能汚染を非常に気にしている。特定の食材については、特別なルートで調達を行っており、子供や妊娠

	who want to get pregnant in the future. I have special purchasing roots for them.	を意識する女性については特別に調達した食材を用いている。
☐	I have a lot of concern about radioactive contamination of food. With respect to some specific food, I try not to give locally produced one to any of my family members. I have special purchasing roots for them.	放射能汚染を非常に気にしている。特定の食材については、特別なルートで調達を行っており、家族全員同じものを食べている。
☐	Others. (Explain explicitly your perception and situation below.)<br><br>____	その他　（具体的に：

Q20 現在お住まいの住宅を選ぶにあたって、あなたのお家にお住まいの方以外の方に相談されましたか。

When you chose to live your current housing, did you consult with anybody who does not live with you?

☐	Yes	☐	No

Q21 あなたが相談された方々を5人思い浮かべてください。その方々のうち、現在仮設住宅に住んでおられる方は何人ですか。

Remember 5 persons you consulted with when you chose to live your current housing. How many of them do live in prefabricated temporary housing provided by the government?

☐	One	☐	Two	☐	Three	☐	Four	☐	Five

Q22 あなたのご家庭の年収（世帯所得）は、税込みでおいくらくらいですか（年金含む）。（ひとつだけ）

How much is the annual income of your household including tax? If you receive a pension, please include it too.

☐.	Less than 2 million yen
☐	More than 2 million yen and less than 4 million yen
☐	More than 4 million yen and less than 6 million yen
☐	More than 6 million yen and less than 8 million yen
☐	More than 8 million yen and less than 10 million yen

☐	More than 10 million yen and less than 15 million yen
☐	More than 15 million yen and less than 20 million yen
☐	More than 20 million yen

Q23 被災当時のあなたのお住まいについて質問します。あなたのお家はいずれですか。（ひとつだけ）

Which type of dwellings did you live in, a single family house or an apartment when Tohoku earthquake happened? Did you rent your dwelling or own it?

	Single family house/Apartment	Renter/Owner	
☐	Single-family house	Renter	一戸建て、賃貸
☐	Single-family house	Owner	一戸建て、持ち家
☐	Apartment, rented	Renter	集合住宅、賃貸
☐	Apartment, rented	Owner	集合住宅、持ち家

【 Q23．で「一戸建て、持ち家」「集合住宅、持ち家」とお答えの方へ 】

If you answered “single-family house and owner”or “apartment and owner”to Q23, please answer Q24.

Q24 被災当時のあなたのお住まいについて、震災に遭われた当時に債務は残っておいででしたか。

Did you have any debt of your home left to pay when Tohoku earthquake occurred?

☐	Yes	☐	No

Q25 あなたが予想する5年後のあなたの収入は、震災以前の何%程度ですか

What percentages of salary that you earned before Tohoku earthquake do you expect you will be able to earn after 5 years?

______________%

Q26 あなたが購読（定期購読である必要はありません）している情報媒体をお答えください。

Name all the information media you buy usually or you used to buy. They do not have to be the ones you regularly subscribe or subscribed.

A．新聞名（新聞名をすべてお答えください）

Name of newspaper (Name all the newspapers you buy)

B.　週刊誌名（週刊誌名をすべてお答えください）

Name of magazines (Name all the magazines you buy)

C.　その他　その他の媒体

Name of other media

Q27 あなたの震災前のお住まいの住所をお答えください。（番地までいただけると大変ありがたく存じますが、町名まででも結構です）

Where did you live before Tohoku earthquake? Please write your address. If possible write a block number as well as a town name.

Q28_1 あなたは第何次の仮設住宅募集に応募されましたか。あてはまるものをすべて選んでください。（第1次から第16次まで）

There were 16 phases of the governmental provision for prefabricated temporary housing. The following table shows the phases and their associated terms. Which phase did you apply for the housing? If you applied for the housing multiple times, check all of the phases you applied.

☐	1st phase	Jan 2011 -	☐	9th phase	Jan 2011 -
☐	2nd phase		☐	10th phase	
☐	3rd phase		☐	11th phase	
☐	4th phase		☐	12th phase	
☐	5th phase		☐	13th phase	

☐	6th phase		☐	14th phase	
☐	7th phase		☐	15th phase	
☐	8th phase		☐	16th phase	

Q28_2 仮設住宅に応募されたのは何月であったか、ご記憶であればご記入ください。

In which month did you apply for the occupancy of prefabricated temporary housing?

Phase you applied				Month			
1st phase	January	Februar y	March				
2nd phase							
3rd phase							
4th phase							
5th phase							
6th phase							
7th phase							
8th phase							
9th phase							
10th phase							
11th phase							
12th phase							
13th phase							
14th phase							
15th phase							
16th phase							Dwf

Q28_3 当選された場合は「当選」に、落選された場合は「落選」を選択してください。（それぞれひとつずつ）

For each of your applications, please indicate whether to win the occupancy of prefabricated temporary housing.

1st phase	□	Won	□	Did not win	9th phase	□	Won	□	Did not win

2nd phase	□	Won	□	Did not win	10th phase	□	Won	□	Did not win
3rd phase	□	Won	□	Did not win	11th phase	□	Won	□	Did not win
4th phase	□	Won	□	Did not win	12th phase	□	Won	□	Did not win
5th phase	□	Won	□	Did not win	13th phase	□	Won	□	Did not win
6th phase	□	Won	□	Did not win	14th phase	□	Won	□	Did not win
7th phase	□	Won	□	Did not win	15th phase	□	Won	□	Did not win
8th phase	□	Won	□	Did not win	16th phase	□	Won	□	Did not win

Q28_4 当選された場合、入居された場合は「入居」に、辞退された場合は「辞退」を選択してください。（それぞれひとつずつ）

If you have a time when you won the occupancy of prefabricated temporary housing but you decided not to live in the prefabricated temporary housing, please indicate when.

□	1st phase	□	5th phase	□	9th phase	□	13th phase
□	2nd phase	□	6th phase	□	10th phase	□	14th phase
□	3rd phase	□	7th phase	□	11th phase	□	15th phase
□	4th phase	□	8th phase	□	12th phase	□	16th phase

Q29 現在あなたのご家族の高齢者の構成についてお伺いします。あなたのお宅に介護の必要な方はおられますか。（ひとつだけ）

Do you currently live with elder people who need nursing care?

☐	Yes	☐	No

Q30 震災以前、あなたはパソコンを持っていましたか。（ひとつだけ）

Did you have a personal computer before Tohoku earthquake occurred?

☐	Yes	☐	No

Q30 震災以前、あなたはインターネットを日にどの程度利用していましたか。（ひとつだけ）

Before Tohoku earthquake occurred, how frequently have you daily used internet service?

☐	I only checked e-mails at a personal computer or a mobile phone.	回数は分からないが、パソコン又は携帯電話でEメールのチェックだけをしていた

☐	I checked news on portal sites such as Yahoo! Japan, MSN Japan, Infoseek, Biglobe and so forth, as well as e-mails at a personal computer or a mobile phone.	E メールのチェックと、ポータルサイト（たとえば Yahoo! Japan や MSN Japan、Infoseek、Biglobe など）でのニュースのチェックをしていた
☐	I checked news on portal sites such as Yahoo! Japan, MSN Japan, Infoseek, Biglobe and so forth, as well as e-mails at a personal computer or a mobile phone. Additionally, I enjoyed netsurfing to collect information.	Eメールのチェックとポータルサイトでのニュースのチェック以外に、ネットサーフィンなどを行って情報収集をしていた
☐	I did not use internet at all.	インターネットは使っていなかった

Q32 あなたは今後以前の居住地に戻ることを計画していますか。（ひとつだけ）

Are you planning to go back to the area you used to live?

☐	Yes	→ Go to Q33 and skip Q34.
☐	No	→ Skip Q33 and go to Q34.

【 Q32. で「はい」とお答えの方へ 】

If you answer “Yes” in Q32, please answer Q33.

Q33 考えられる理由を上位３つまで選んでください

The following list is the reasons making you to plan to go back to the area you used to live. The list consists of 6 reasons; (a), (b), (c), (d), (e) and (f). Choose the applicable 3 reasons for you from them, and then prioritize them. You can find a bracket left to each reason. Write “1”, “2” and “3” in brackets of the firstly, the secondly, and the thirdly prioritized reasons, respectively.

[ ]	(a)	The area where I used to live is the area where I grew up. The area is not very devastated and thus it is possible to go back there.	自分が生まれ育った土地であり、戻ることが可能である
[ ]	(b)	People who my family members know very well in the community of the area where I lived, such as relatives, friends, colleagues from their working places, will go back to the area.	自分と関わりが深い人たちも、そこに戻ることが分かっている

[ ]	(c)	I have my assets and fundamentals of my job in the area.	自分の資産や生業の基盤がある
[ ]	(d)	I do not think that I can fit in a new community in different places from the place where I used to live.	新しい土地での生活はなじめそうにない
[ ]	(e)	I would like to help the recovery of the community of the area.	復興を自分の手で成功させたい
[ ]	(f)	Other reasons	その他

Q33SQ1 Q33で「その他」とご回答の方は具体的にご記入ください。

If you include “Others reasons” in your prioritized 3 reasons in Q33, please explain it explicitly below.

【 Q32. で「いいえ」とお答えの方へ 】

If you answer “No” in Q32, please answer Q34.

Q34 考えられる理由を上位３つまで選んでください。（上位から順番にお答えください）

The following list is the reasons preventing you from planning to go back to the area you used to live. The list consists of 5 reasons; (a), (b), (c), (d) and (e). Choose the applicable 3 reasons for you from them, and then prioritize them. You can find a bracket left to each reason. Write “1”, “2” and “3” in brackets of the firstly, the secondly, and the thirdly prioritized reasons, respectively.

[ ]	(a)	The area where I used to live is the area where I grew up. The area is not very devastated and thus it is possible to go back there.	自分が生まれ育った土地だが、戻ることが不可能である
[ ]	(b)	People who my family members know very well in the community of the area, such as relatives, friends, colleagues from their working places, may not go back to the area.	自分と関わりが深い人たちが、そこに戻ることが不明である
[ ]	(c)	I do not have my assets and fundamentals of my job.in the area	自分の資産や生業の基盤がない

[ ]	(d)	I would like to start new life in a new place so that I can keep distance from the memory of Tohoku earthquake area.	震災の記憶から身を離して、新しい土地での生活を始めたい
[ ]	(e)	Other reasons	その他

Q34SQ1 Q34で「その他」とご回答の方は具体的にご記入ください。

If you include "Others reasons" in your prioritized 3 reasons in Q34, please explain it explicitly below.

____
____
____
