# Supplementary material for: The Effects of Community Attachment and Information Seeking on Displaced Disaster Victims’ Decision Making
Source: PLoS One. 2016 Mar 23;11(3):e0151928. doi: 10.1371/journal.pone.0151928 (PMC4805184; doi:10.1371/journal.pone.0151928)
Supplement: S1 Internet Survey — Japanese only. (PDF) [file pone.0151928.s005.pdf]

(pid)

「世界に貢献する環境経済の政策研究」における生物多様性とコミュニティーとの関わりの調査

【当ＷＥＢシステム利用上の注意】

再ログイン時には下記ＩＤ番号、パスワードが必要です。
＜ＩＤ番号、パスワードをメモしておくことをお薦めします＞

**ＩＤ番号：**	$Aid
**パスワード：**	$Af

- 回答中に前ページに戻る際には、ページの一番下の「戻る」ボタンをクリックしてください。ブラウザの「戻る」ボタンは使用しないようご注意ください。
- 回答期間中は、土日も含め24時間回答可能です。
- 推奨ブラウザはWindowsでは「InternetExplorer6,7,8」「firefox 3.5」です。これ以外のブラウザでは回答時にエラーが出る可能性があります。
- 一度、回答を完了すると再回答はできませんのでご注意下さい。
- 回線の混雑などが原因で反応が遅くなることがあります。ご了承ください。

次へ ▶

（ファイル名 iissue01.htm）

- 回答内容により質問番号が飛ぶことがありますが、気にせずにご回答ください。
- 回線の混雑などが原因で反応が遅くなることがあります。ご了承ください。
- ブラウザの戻るボタンで戻った場合、回答が正しく受け付けられない場合があります。その場合、再度アンケートを最初からやり直して下さい。

（調査NO.12-811-0045）

NIKKEI-R

(pid)

「世界に貢献する環境経済の政策研究」における生物多様性とコミュニティーとの関わりの調査

S1

あなたのお住まいの都道府県は。　《ひとつだけ》

(s01)=　▽以下から選択

(s01)=01　北海道

(s01)=02　青森県

(s01)=03　岩手県

(s01)=04　宮城県

(s01)=05 秋田県

(s01)=06 山形県

(s01)=07 福島県

(s01)=08 茨城県

(s01)=09 栃木県

(s01)=10 群馬県

(s01)=11 埼玉県

(s01)=12 千葉県

(s01)=13 東京都

(s01)=14 神奈川県

(s01)=15 新潟県

(s01)=16 富山県

(s01)=17 石川県

(s01)=18 福井県

(s01)=19 山梨県

(s01)=20 長野県

(s01)=21 岐阜県

(s01)=22 静岡県

(s01)=23 愛知県

(s01)=24 三重県

(s01)=25 滋賀県

(s01)=26 京都府

(s01)=27 大阪府

(s01)=28 兵庫県

(s01)=29 奈良県

(s01)=30 和歌山県

(s01)=31 鳥取県

(s01)=32 島根県

(s01)=33 岡山県

(s01)=34 広島県

(s01)=35 山口県

(s01)=36 徳島県

(s01)=37　香川県

(s01)=38　愛媛県

(s01)=39　高知県

(s01)=40　福岡県

(s01)=41　佐賀県

(s01)=42　長崎県

(s01)=43　熊本県

(s01)=44　大分県

(s01)=45　宮崎県

(s01)=46　鹿児島県

(s01)=47　沖縄県

S2

東日本大震災で、あなたの生活には以下のような影響がありましたか。　《いくつでも》

(s02_01)=1 □ 避難所生活を経験した
(s02_02)=1 □ 自宅を移転した
(s02_03)=1 □ 停電（計画停電でない）を経験した
(s02_04)=1 □ 計画停電を経験した
(s02_05)=1 □ 断水を経験した
(s02_06)=1 □ ガスが使えない状態を経験した
(s02_07)=1 □ 帰宅難民になった
(s02_08)=1 □ 水を買いだめした
(s02_09)=1 □ お米を買いだめした
(s02_10)=1 □ 節電のために何かを購入した
(s02_11)=1 □ 上記以外のことを経験した
(s02_12)=1 □ 震災による影響は特になかった

▶ このページに入力したものを全てクリアする

次へ ▶

（ファイル名　s01.htm）

- 回答内容により質問番号が飛ぶことがありますが、気にせずにご回答ください。

- 回線の混雑などが原因で反応が遅くなることがあります。ご了承ください。
- ブラウザの戻るボタンで戻った場合、回答が正しく受け付けられない場合があります。その場合、再度アンケートを最初からやり直して下さい。

（調査NO. 12-811-0045）

NIKKEI-R

(pid)

「世界に貢献する環境経済の政策研究」における生物多様性とコミュニティーとの関わりの調査

Q1_1

あなたの性別をお答えください。 《ひとつだけ》

(q01_1)=1 男性
(q01_1)=2 女性

Q1_2

あなたの年齢をお答えください。あなたはお幾つですか。 《ひとつだけ》

(q01_2)= ▽以下から選択
(q01_2)=15 15歳以下
(q01_2)=16 16歳
(q01_2)=17 17歳
(q01_2)=18 18歳
(q01_2)=19 19歳
(q01_2)=20 20歳
(q01_2)=21 21歳
(q01_2)=22 22歳
(q01_2)=23 23歳
(q01_2)=24 24歳
(q01_2)=25 25歳
(q01_2)=26 26歳
(q01_2)=27 27歳
(q01_2)=28 28歳
(q01_2)=29 29歳

(q01_2)=30 30歳

(q01_2)=31 31歳

(q01_2)=32 32歳

(q01_2)=33 33歳

(q01_2)=34 34歳

(q01_2)=35 35歳

(q01_2)=36 36歳

(q01_2)=37 37歳

(q01_2)=38 38歳

(q01_2)=39 39歳

(q01_2)=40 40歳

(q01_2)=41 41歳

(q01_2)=42 42歳

(q01_2)=43 43歳

(q01_2)=44 44歳

(q01_2)=45 45歳

(q01_2)=46 46歳

(q01_2)=47 47歳

(q01_2)=48 48歳

(q01_2)=49 49歳

(q01_2)=50 50歳

(q01_2)=51 51歳

(q01_2)=52 52歳

(q01_2)=53 53歳

(q01_2)=54 54歳

(q01_2)=55 55歳

(q01_2)=56 56歳

(q01_2)=57 57歳

(q01_2)=58 58歳

(q01_2)=59 59歳

(q01_2)=60 60歳

(q01_2)=61 61歳

(q01_2)=62 62歳

(q01_2)=63 63歳

(q01_2)=64 64歳

(q01_2)=65 65歳

(q01_2)=66 66歳

(q01_2)=67 67歳

(q01_2)=68 68歳

(q01_2)=69 69歳

(q01_2)=70 70歳以上

Q2

あなたが現在お住まいの住宅は、震災前のあなたのお家と比べてどちらにありますか。 《ひとつだけ》

(q02)=1 同じ県の同じ市内

(q02)=2 同じ県の市外

(q02)=3 他県

Q2SQ1

あなたは過去に仮設住宅に応募された経験がありますか。 《ひとつだけ》

(q02sq1)=1 ある

(q02sq1)=2 ない

このページに入力したものを全てクリアする

戻る 次へ

（ファイル名 q01_1.htm）

- 回答内容により質問番号が飛ぶことがありますが、気にせずにご回答ください。
- 回線の混雑などが原因で反応が遅くなることがあります。ご了承ください。
- ブラウザの戻るボタンで戻った場合、回答が正しく受け付けられない場合があります。その場合、再度アンケートを最初からやり直して下さい。

（調査NO. 12-811-0045）

(pid)

「世界に貢献する環境経済の政策研究」における生物多様性とコミュニティーとの関わりの調査

Q3

あなたが仮設住宅入居に応募したのはいつのことですか。現在仮設住宅にお住まいでない方は、仮設住宅入居に応募された経験がある場合お答えください。

(q03_1)= –
(q03_1)=01 1
(q03_1)=02 2
(q03_1)=03 3
(q03_1)=04 4
(q03_1)=05 5
(q03_1)=06 6
(q03_1)=07 7
(q03_1)=08 8
(q03_1)=09 9
(q03_1)=10 10
(q03_1)=11 11
(q03_1)=12 12

月

(q03_2)= –
(q03_2)=01 1
(q03_2)=02 2
(q03_2)=03 3
(q03_2)=04 4
(q03_2)=05 5
(q03_2)=06 6
(q03_2)=07 7
(q03_2)=08 8
(q03_2)=09 9
(q03_2)=10 10
(q03_2)=11 11
(q03_2)=12 12
(q03_2)=13 13
(q03_2)=14 14
(q03_2)=15 15
(q03_2)=16 16
(q03_2)=17 17
(q03_2)=18 18
(q03_2)=19 19
(q03_2)=20 20
(q03_2)=21 21
(q03_2)=22 22
(q03_2)=23 23
(q03_2)=24 24
(q03_2)=25 25
(q03_2)=26 26
(q03_2)=27 27

日

(q03_2)=28 28

(q03_2)=29 29

(q03_2)=30 30

(q03_2)=31 31

(xq03)

Q4

あなたは現在の住宅に入居する前に当選した仮設住宅への入居を辞退されたことはありますか。 《ひとつだけ》

(q04)=1 はい

(q04)=2 いいえ

このページに入力したものを全てクリアする

戻る 次へ

(ファイル名 q03.htm)

- 回答内容により質問番号が飛ぶことがありますが、気にせずにご回答ください。
- 回線の混雑などが原因で反応が遅くなることがあります。ご了承ください。
- ブラウザの戻るボタンで戻った場合、回答が正しく受け付けられない場合があります。その場合、再度アンケートを最初からやり直して下さい。

(調査NO. 12-811-0045)

(pid)

「世界に貢献する環境経済の政策研究」における生物多様性とコミュニティーとの関わりの調査

Q5

その理由は次のいずれですか。 《いくつでも》

(q05_1)=1 当選した仮設住宅の位置が、被災した当時の家と似ていた（海に近いなど）

(q05_2)=1 当選した仮設住宅が自分の職場から遠すぎた

(q05_3)=1 当選した仮設住宅では、家族のための医療のケアが難しかった

(q05_4)=1 当選した仮設住宅で、新しいコミュニティーに参加することに不安があった

(q05_5)=1 辞退した時点では、生活をはじめるための経済的な条件があなた自身にそろっていなかった
（金銭面で生活を始める条件が揃っていなかった）

(q05_6)=1 辞退した時点では、生活をはじめるための経済的な条件が周囲の環境にそろっていなかった
（物を調達するための施設が近くに揃っていなかった）

(q05_7)=1 その他 （具体的に： (q05_7x) ）

Q6

あなたのご家庭の現在同居している方の人数は、あなたを含めて何人おられますか。 （ひとつだけ）

(q06)=1 １人
(q06)=2 ２人
(q06)=3 ３人
(q06)=4 ４人
(q06)=5 ５人
(q06)=6 ６人
(q06)=7 ７人以上

Q7

あなたのご家族に、65歳以上の方は何人おられますか。 （ひとつだけ）

(q07)=0 ０人
(q07)=1 １人
(q07)=2 ２人
(q07)=3 ３人以上

このページに入力したものを全てクリアする

戻る 次へ

（ファイル名 q05.htm）

- 回答内容により質問番号が飛ぶことがありますが、気にせずにご回答ください。
- 回線の混雑などが原因で反応が遅くなることがあります。ご了承ください。
- ブラウザの戻るボタンで戻った場合、回答が正しく受け付けられない場合があります。その場合、再度アンケートを最初からやり直して下さい。

（調査NO. 12-811-0045）

NIKKEI-R

(pid)

「世界に貢献する環境経済の政策研究」における生物多様性とコ

ミュニティーとの関わりの調査

Q8

あなたのご家族に、18歳以下で教育機関に通われている方は何人おられますか。 （ひとつだけ）

(q08)=0 ０人
(q08)=1 １人
(q08)=2 ２人
(q08)=3 ３人以上

Q9

このうち、被扶養者（主に生計を支える人以外の人）は何人ですか。 （ひとつだけ）

(q09)=1 １人
(q09)=2 ２人
(q09)=3 ３人
(q09)=4 ４人
(q09)=5 ５人
(q09)=6 ６人以上

このページに入力したものを全てクリアする

戻る　次へ

（ファイル名 q08.htm）

- 回答内容により質問番号が飛ぶことがありますが、気にせずにご回答ください。
- 回線の混雑などが原因で反応が遅くなることがあります。ご了承ください。
- ブラウザの戻るボタンで戻った場合、回答が正しく受け付けられない場合があります。その場合、再度アンケートを最初からやり直して下さい。

（調査NO. 12-811-0045）

NIKKEI-R

(pid)

「世界に貢献する環境経済の政策研究」における生物多様性とコミュニティーとの関わりの調査

Q10

あなたのご職業は次のどちらですか。 （ひとつだけ）

(q10)=01 会社員
(q10)=02 農林業（専業）
(q10)=03 漁業（専業）
(q10)=04 自営業
(q10)=05 公務員・教職員
(q10)=06 会社員と農林又は漁業との兼業
(q10)=07 自営業と農林又は漁業との兼業
(q10)=08 公務員・教職員と農林又は漁業との兼業
(q10)=09 パート・アルバイト
(q10)=10 学生
(q10)=11 専業主婦
(q10)=12 年金生活
(q10)=13 無職
(q10)=14 その他 （具体的に：(q10_14x)　）

Q11

あなたが震災以前に住んでおられた土地に、震災に遭われるまで何年お住まいでしたか。 《ひとつだけ》

(q11)= ▽以下から選択
(q11)=00 0年
(q11)=01 1年
(q11)=02 2年
(q11)=03 3年
(q11)=04 4年
(q11)=05 5年
(q11)=06 6年
(q11)=07 7年
(q11)=08 8年
(q11)=09 9年
(q11)=10 10年
(q11)=11 11年
(q11)=12 12年

(q11)=13 13年

(q11)=14 14年

(q11)=15 15年

(q11)=16 16年

(q11)=17 17年

(q11)=18 18年

(q11)=19 19年

(q11)=20 20年

(q11)=21 21年

(q11)=22 22年

(q11)=23 23年

(q11)=24 24年

(q11)=25 25年

(q11)=26 26年

(q11)=27 27年

(q11)=28 28年

(q11)=29 29年

(q11)=30 30年

(q11)=31 31年

(q11)=32 32年

(q11)=33 33年

(q11)=34 34年

(q11)=35 35年

(q11)=36 36年

(q11)=37 37年

(q11)=38 38年

(q11)=39 39年

(q11)=40 40年

(q11)=41 41年

(q11)=42 42年

(q11)=43 43年

(q11)=44 44年

(q11)=45 45年

(q11)=46 46年

(q11)=47 47年

(q11)=48 48年

(q11)=49 49年

(q11)=50 50年

(q11)=51 51年

(q11)=52 52年

(q11)=53 53年

(q11)=54 54年

(q11)=55 55年

(q11)=56 56年

(q11)=57 57年

(q11)=58 58年

(q11)=59 59年

(q11)=60 60年

(q11)=61 61年

(q11)=62 62年

(q11)=63 63年

(q11)=64 64年

(q11)=65 65年

(q11)=66 66年

(q11)=67 67年

(q11)=68 68年

(q11)=69 69年

(q11)=70 70年

(q11)=71 71年以上

Q12

あなたが震災以前に住んでおられた土地には、ご親族のうちであなたが初めての代ですか。 《ひとつだけ》

(q12)=1 はい

(q12)=2 いいえ

このページに入力したものを全てクリアする

戻る　次へ

（ファイル名 q110.htm）

- 回答内容により質問番号が飛ぶことがありますが、気にせずにご回答ください。
- 回線の混雑などが原因で反応が遅くなることがあります。ご了承ください。
- ブラウザの戻るボタンで戻った場合、回答が正しく受け付けられない場合があります。その場合、再度アンケートを最初からやり直して下さい。

（調査NO. 12-811-0045）

NIKKEI-R

(pid)

「世界に貢献する環境経済の政策研究」における生物多様性とコミュニティーとの関わりの調査

Q13

あなたが震災以前に住んでおられた土地には、あなたはご親族の代から合計で何年お住まいでしたか。

《ひとつだけ》

(q13)= ▽以下から選択

(q13)=000 0年

(q13)=001 1年

(q13)=002 2年

(q13)=003 3年

(q13)=004 4年

(q13)=005 5年

(q13)=006 6年

(q13)=007 7年

(q13)=008 8年

(q13)=009 9年

(q13)=010 10年

(q13)=011 11年

(q13)=012 12年

(q13)=013 13年

(q13)=014 14年

(q13)=015 15年

(q13)=016 16年

(q13)=017 17年

(q13)=018 18年

(q13)=019 19年

(q13)=020 20年

(q13)=021 21年

(q13)=022 22年

(q13)=023 23年

(q13)=024 24年

(q13)=025 25年

(q13)=026 26年

(q13)=027 27年

(q13)=028 28年

(q13)=029 29年

(q13)=030 30年

(q13)=031 31年

(q13)=032 32年

(q13)=033 33年

(q13)=034 34年

(q13)=035 35年

(q13)=036 36年

(q13)=037 37年

(q13)=038 38年

(q13)=039 39年

(q13)=040 40年

(q13)=041 41年

(q13)=042 42年

(q13)=043 43年

(q13)=044 44年

(q13)=045 45年

(q13)=046 46年

(q13)=047 47年

(q13)=048 48年

(q13)=049 49年

(q13)=050 50年

(q13)=051 51年

(q13)=052 52年

(q13)=053 53年

(q13)=054 54年

(q13)=055 55年

(q13)=056 56年

(q13)=057 57年

(q13)=058 58年

(q13)=059 59年

(q13)=060 60年

(q13)=061 61年

(q13)=062 62年

(q13)=063 63年

(q13)=064 64年

(q13)=065 65年

(q13)=066 66年

(q13)=067 67年

(q13)=068 68年

(q13)=069 69年

(q13)=070 70年

(q13)=071 71年

(q13)=072 72年

(q13)=073 73年

(q13)=074 74年

(q13)=075 75年

(q13)=076 76年

(q13)=077 77年

(q13)=078 78年

(q13)=079 79年

(q13)=080 80年

(q13)=081 81年

(q13)=082 82年

(q13)=083 83年

(q13)=084 84年

(q13)=085 85年

(q13)=086 86年

(q13)=087 87年

(q13)=088 88年

(q13)=089 89年

(q13)=090 90年

(q13)=091 91年

(q13)=092 92年

(q13)=093 93年

(q13)=094 94年

(q13)=095 95年

(q13)=096 96年

(q13)=097 97年

(q13)=098 98年

(q13)=099 99年

(q13)=100 100年

(q13)=101 101年

(q13)=102 102年

(q13)=103 103年

(q13)=104 104年

(q13)=105 105年

(q13)=106 106年

(q13)=107 107年

(q13)=108 108年

(q13)=109 109年

(q13)=110 110年

(q13)=111 111年

(q13)=112 112年

(q13)=113 113年

(q13)=114 114年

(q13)=115 115年

(q13)=116 116年

(q13)=117 117年

(q13)=118 118年

(q13)=119 119年

(q13)=120 120年

(q13)=121 121年

(q13)=122 122年

(q13)=123 123年

(q13)=124 124年

(q13)=125 125年

(q13)=126 126年

(q13)=127 127年

(q13)=128 128年

(q13)=129 129年

(q13)=130 130年

(q13)=131 131年

(q13)=132 132年

(q13)=133 133年

(q13)=134 134年

(q13)=135 135年

(q13)=136 136年

(q13)=137 137年

(q13)=138 138年

(q13)=139 139年

(q13)=140　140年

(q13)=141　141年

(q13)=142　142年

(q13)=143　143年

(q13)=144　144年

(q13)=145　145年

(q13)=146　146年

(q13)=147　147年

(q13)=148　148年

(q13)=149　149年

(q13)=150　150年

(q13)=151　151年

(q13)=152　152年

(q13)=153　153年

(q13)=154　154年

(q13)=155　155年

(q13)=156　156年

(q13)=157　157年

(q13)=158　158年

(q13)=159　159年

(q13)=160　160年

(q13)=161　161年

(q13)=162　162年

(q13)=163　163年

(q13)=164　164年

(q13)=165　165年

(q13)=166　166年

(q13)=167　167年

(q13)=168　168年

(q13)=169　169年

(q13)=170　170年

(q13)=171 171年

(q13)=172 172年

(q13)=173 173年

(q13)=174 174年

(q13)=175 175年

(q13)=176 176年

(q13)=177 177年

(q13)=178 178年

(q13)=179 179年

(q13)=180 180年

(q13)=181 181年

(q13)=182 182年

(q13)=183 183年

(q13)=184 184年

(q13)=185 185年

(q13)=186 186年

(q13)=187 187年

(q13)=188 188年

(q13)=189 189年

(q13)=190 190年

(q13)=191 191年

(q13)=192 192年

(q13)=193 193年

(q13)=194 194年

(q13)=195 195年

(q13)=196 196年

(q13)=197 197年

(q13)=198 198年

(q13)=199 199年

(q13)=200 200年

(q13)=201 201年以上

Q14

震災以前、あなたのご家族に消防団に所属している方は何人おられましたか。 （人数は右づめでご記入ください）

(q14_2)=	–	(q14_1)=	–
(q14_2)=0	0	(q14_1)=0	0
(q14_2)=1	1	(q14_1)=1	1
(q14_2)=2	2	(q14_1)=2	2
(q14_2)=3	3	(q14_1)=3	3
(q14_2)=4	4	(q14_1)=4	4 人
(q14_2)=5	5	(q14_1)=5	5
(q14_2)=6	6	(q14_1)=6	6
(q14_2)=7	7	(q14_1)=7	7
(q14_2)=8	8	(q14_1)=8	8
(q14_2)=9	9	(q14_1)=9	9

(xq14)

このページに入力したものを全てクリアする

戻る　次へ

（ファイル名 q13.htm）

- 回答内容により質問番号が飛ぶことがありますが、気にせずにご回答ください。
- 回線の混雑などが原因で反応が遅くなることがあります。ご了承ください。
- ブラウザの戻るボタンで戻った場合、回答が正しく受け付けられない場合があります。その場合、再度アンケートを最初からやり直して下さい。

（調査NO. 12-811-0045）

(pid)

「世界に貢献する環境経済の政策研究」における生物多様性とコミュニティーとの関わりの調査

Q15

あなたが消防団以外の地域の活動（祭やイベントなど）に参加する頻度はどの程度ですか。 （ひとつだけ）

(q15)=1 積極的に参加していて、古参の人と話をする機会がある

(q15)=2 参加しているが、古参の人と話をするほどではない
(q15)=3 あまり参加しない

Q16

あなたが仮設住宅入居に応募したとき、重視されたことを3つお答えください。

（半角カタカナは使用しないでください）

＜１つ目＞ (q16x_1)
＜２つ目＞ (q16x_2)
＜３つ目＞ (q16x_3)

このページに入力したものを全てクリアする

戻る 次へ

（ファイル名 q15.htm）

- 回答内容により質問番号が飛ぶことがありますが、気にせずにご回答ください。
- 回線の混雑などが原因で反応が遅くなることがあります。ご了承ください。
- ブラウザの戻るボタンで戻った場合、回答が正しく受け付けられない場合があります。その場合、再度アンケートを最初からやり直して下さい。

（調査NO. 12-811-0045）

NIKKEI-R

(pid)

「世界に貢献する環境経済の政策研究」における生物多様性とコミュニティーとの関わりの調査

Q17

東北地方太平洋沖地震（2011年3月11日に発生した地震）以外に、地震以外を含む（台風など）あなたの記憶に強く印象に残っている災害を5つお答えください。正確な名称である必要はありません。

（半角カタカナは使用しないでください）

① (q17x_1)
② (q17x_2)
③ (q17x_3)
④ (q17x_4)
⑤ (q17x_5)

Q18

Q17. でお答えになった災害のうち、実際に体験されたものはどれですか。

（いくつでも）

(q18_1)=1 ①

(q18_2)=1 ②

(q18_3)=1 ③

(q18_4)=1 ④

(q18_5)=1 ⑤

(q18_6)=1 いずれも実際には体験していない

このページに入力したものを全てクリアする

戻る 次へ

（ファイル名 q17.htm）

- 回答内容により質問番号が飛ぶことがありますが、気にせずにご回答ください。
- 回線の混雑などが原因で反応が遅くなることがあります。ご了承ください。
- ブラウザの戻るボタンで戻った場合、回答が正しく受け付けられない場合があります。その場合、再度アンケートを最初からやり直して下さい。

（調査NO. 12-811-0045）

(pid)

「世界に貢献する環境経済の政策研究」における生物多様性とコミュニティーとの関わりの調査

Q19

放射能汚染への意識と食事の関係についてご質問します。あなたのお家の食事では、次のうちどのような形態をとっておられますか。 （ひとつだけ）

(q19)=1 放射能汚染は全く気にしていない。食材は可能な限り地産地消を意識して購入している。

(q19)=2 放射能汚染は少し気にしているが、特別なルートで食材を調達してはいない。

(q19)=3 放射能汚染は少し気にしているが、特別なルートで食材を調達できない。

(q19)=4 放射能汚染は少し気にしている。特定の食材については、特別なルートで調達を行い、子供や妊娠を意識する女性については特別に調達した食材を用いている。

(q19)=5 放射能汚染は少し気にしている。特定の食材については、特別なルートで調達を行い、家族全員同じものを食べている。

(q19)=6 放射能汚染を非常に気にしているが、特別なルートで食材を調達できない。

(q19)=7 放射能汚染を非常に気にしている。特定の食材については、特別なルートで調達を行っており、子供や妊娠を意識する女性については特別に調達した食材を用いている。

(q19)=8 放射能汚染を非常に気にしている。特定の食材については、特別なルートで調達を行っており、家族全員同じものを食べている。

(q19)=9 その他 （具体的に： (q19_9x) ）

Q20

現在お住まいの住宅を選ぶにあたって、あなたのお家にお住まいの方以外の方に相談されましたか。 （ひとつだけ）

(q20)=1 はい

(q20)=2 いいえ

このページに入力したものを全てクリアする

戻る 次へ

（ファイル名 q19.htm）

- 回答内容により質問番号が飛ぶことがありますが、気にせずにご回答ください。
- 回線の混雑などが原因で反応が遅くなることがあります。ご了承ください。
- ブラウザの戻るボタンで戻った場合、回答が正しく受け付けられない場合があります。その場合、再度アンケートを最初からやり直して下さい。

（調査NO. 12-811-0045）

NIKKEI-R

(pid)

「世界に貢献する環境経済の政策研究」における生物多様性とコミュニティーとの関わりの調査

【 Q20. で「はい」とお答えの方へ 】

Q21

あなたが相談された方々を5人思い浮かべてください。その方々のうち、現在仮設住宅に住んでおられる方は何人ですか。 （ひとつだけ）

(q21)=1 １人

(q21)=2 ２人

(q21)=3 ３人

(q21)=4 ４人

(q21)=5 ５人

Q22

あなたのご家庭の年収（世帯所得）は、税込みでおいくらくらいですか（年金含む）。 （ひとつだけ）

(q22)=1 200万円未満

(q22)=2 200万円～400万円未満

(q22)=3 400万円～600万円未満
(q22)=4 600万円～800万円未満
(q22)=5 800万円～1000万円未満
(q22)=6 1000万円～1500万円未満
(q22)=7 1500万円～2000万円未満
(q22)=8 2000万円以上

Q23

被災当時のあなたのお住まいについて質問します。あなたのお家はいずれですか。 （ひとつだけ）

(q23)=1 一戸建て、賃貸
(q23)=2 一戸建て、持ち家
(q23)=3 集合住宅、賃貸
(q23)=4 集合住宅、持ち家

このページに入力したものを全てクリアする

戻る　次へ

（ファイル名 q21..htm）

- 回答内容により質問番号が飛ぶことがありますが、気にせずにご回答ください。
- 回線の混雑などが原因で反応が遅くなることがあります。ご了承ください。
- ブラウザの戻るボタンで戻った場合、回答が正しく受け付けられない場合があります。その場合、再度アンケートを最初からやり直して下さい。

（調査NO. 12-811-0045）

NIKKEI-R

(pid)

「世界に貢献する環境経済の政策研究」における生物多様性とコミュニティーとの関わりの調査

【 Q23. で「一戸建て、持ち家」「集合住宅、持ち家」とお答えの方へ 】

Q24

被災当時のあなたのお住まいについて、震災に遭われた当時に債務は残っておいででしたか。 （ひとつだけ）

(q24)=1 はい
(q24)=2 いいえ

Q25

あなたが予想する5年後のあなたの収入は、震災以前の何%程度ですか。 (右づめでご記入ください)

(q25_3)=	–	(q25_2)=	–	(q25_1)=	–
(q25_3)=0	0	(q25_2)=0	0	(q25_1)=0	0
(q25_3)=1	1	(q25_2)=1	1	(q25_1)=1	1
(q25_3)=2	2	(q25_2)=2	2	(q25_1)=2	2
(q25_3)=3	3	(q25_2)=3	3	(q25_1)=3	3
(q25_3)=4	4	(q25_2)=4	4	(q25_1)=4	4 %
(q25_3)=5	5	(q25_2)=5	5	(q25_1)=5	5
(q25_3)=6	6	(q25_2)=6	6	(q25_1)=6	6
(q25_3)=7	7	(q25_2)=7	7	(q25_1)=7	7
(q25_3)=8	8	(q25_2)=8	8	(q25_1)=8	8
(q25_3)=9	9	(q25_2)=9	9	(q25_1)=9	9

(xq25)

Q26

あなたが購読（定期購読である必要はありません）している情報媒体をお答えください。 (半角カタカナは使用しないでください。)

A. 新聞名（新聞名をすべてお答えください）

(q26x_1)

B. 週刊誌名（週刊誌名をすべてお答えください）

(q26x_2)

C. その他　その他の媒体

(q26x_4)

Q27

あなたの震災前のお住まいの住所をお答えください。 (半角カタカナは使用しないでください)

（番地までいただけると大変ありがたく存じますが、町名まででも結構です）

(q27x) [ ]

このページに入力したものを全てクリアする

戻る 次へ

（ファイル名 q24.htm）

- 回答内容により質問番号が飛ぶことがありますが、気にせずにご回答ください。
- 回線の混雑などが原因で反応が遅くなることがあります。ご了承ください。
- ブラウザの戻るボタンで戻った場合、回答が正しく受け付けられない場合があります。その場合、再度アンケートを最初からやり直して下さい。

（調査NO. 12-811-0045）

NIKKEI-R

(pid)

「世界に貢献する環境経済の政策研究」における生物多様性とコミュニティーとの関わりの調査

Q28_1

あなたは第何次の仮設住宅募集に応募されましたか。あてはまるものをすべて選んでください。 《いくつでも》

(q28_1_01)=1 第1次	(q28_1_05)=1 第5次	(q28_1_09)=1 第9次	(q28_1_13)=1 第13次
(q28_1_02)=1 第2次	(q28_1_06)=1 第6次	(q28_1_10)=1 第10次	(q28_1_14)=1 第14次
(q28_1_03)=1 第3次	(q28_1_07)=1 第7次	(q28_1_11)=1 第11次	(q28_1_15)=1 第15次
(q28_1_04)=1 第4次	(q28_1_08)=1 第8次	(q28_1_12)=1 第12次	(q28_1_16)=1 第16次

このページに入力したものを全てクリアする

戻る 次へ

（ファイル名 q28_1.htm）

- 回答内容により質問番号が飛ぶことがありますが、気にせずにご回答ください。
- 回線の混雑などが原因で反応が遅くなることがあります。ご了承ください。
- ブラウザの戻るボタンで戻った場合、回答が正しく受け付けられない場合があります。その場合、再度アンケートを最初からやり直して下さい。

（調査NO.12-811-0045）

(pid)

「世界に貢献する環境経済の政策研究」における生物多様性とコミュニティーとの関わりの調査

Q28_2

仮設住宅に応募されたのは何月であったか、ご記憶であればご記入ください。　《ひとつだけ》

【第1次】

(q28_2_1)=　▽以下から選択
(q28_2_1)=01　1月
(q28_2_1)=02　2月
(q28_2_1)=03　3月
(q28_2_1)=04　4月
(q28_2_1)=05　5月
(q28_2_1)=06　6月
(q28_2_1)=07　7月
(q28_2_1)=08　8月
(q28_2_1)=09　9月
(q28_2_1)=10　10月
(q28_2_1)=11　11月
(q28_2_1)=12　12月

【第2次】

(q28_2_2)=　▽以下から選択
(q28_2_2)=01　1月
(q28_2_2)=02　2月
(q28_2_2)=03　3月

(q28_2_2)=04 4月

(q28_2_2)=05 5月

(q28_2_2)=06 6月

(q28_2_2)=07 7月

(q28_2_2)=08 8月

(q28_2_2)=09 9月

(q28_2_2)=10 10月

(q28_2_2)=11 11月

(q28_2_2)=12 12月

【第3次】

(q28_2_3)= ▽以下から選択

(q28_2_3)=01 1月

(q28_2_3)=02 2月

(q28_2_3)=03 3月

(q28_2_3)=04 4月

(q28_2_3)=05 5月

(q28_2_3)=06 6月

(q28_2_3)=07 7月

(q28_2_3)=08 8月

(q28_2_3)=09 9月

(q28_2_3)=10 10月

(q28_2_3)=11 11月

(q28_2_3)=12 12月

【第4次】

(q28_2_4)= ▽以下から選択

(q28_2_4)=01 1月

(q28_2_4)=02 2月

(q28_2_4)=03 3月

(q28_2_4)=04 4月

(q28_2_4)=05 5月

(q28_2_4)=06 6月

(q28_2_4)=07 7月

(q28_2_4)=08 8月

(q28_2_4)=09 9月

(q28_2_4)=10 10月

(q28_2_4)=11 11月

(q28_2_4)=12 12月

【第5次】

(q28_2_5)= ▽以下から選択

(q28_2_5)=01 1月

(q28_2_5)=02 2月

(q28_2_5)=03 3月

(q28_2_5)=04 4月

(q28_2_5)=05 5月

(q28_2_5)=06 6月

(q28_2_5)=07 7月

(q28_2_5)=08 8月

(q28_2_5)=09 9月

(q28_2_5)=10 10月

(q28_2_5)=11 11月

(q28_2_5)=12 12月

【第6次】

(q28_2_6)= ▽以下から選択

(q28_2_6)=01　1月

(q28_2_6)=02　2月

(q28_2_6)=03　3月

(q28_2_6)=04　4月

(q28_2_6)=05　5月

(q28_2_6)=06　6月

(q28_2_6)=07　7月

(q28_2_6)=08　8月

(q28_2_6)=09　9月

(q28_2_6)=10　10月

(q28_2_6)=11　11月

(q28_2_6)=12　12月

【第7次】

(q28_2_7)=　▽以下から選択

(q28_2_7)=01　1月

(q28_2_7)=02　2月

(q28_2_7)=03　3月

(q28_2_7)=04　4月

(q28_2_7)=05　5月

(q28_2_7)=06　6月

(q28_2_7)=07　7月

(q28_2_7)=08　8月

(q28_2_7)=09　9月

(q28_2_7)=10　10月

(q28_2_7)=11　11月

(q28_2_7)=12　12月

【第8次】

(q28_2_8)=　▽以下から選択
(q28_2_8)=01　1月
(q28_2_8)=02　2月
(q28_2_8)=03　3月
(q28_2_8)=04　4月
(q28_2_8)=05　5月
(q28_2_8)=06　6月
(q28_2_8)=07　7月
(q28_2_8)=08　8月
(q28_2_8)=09　9月
(q28_2_8)=10　10月
(q28_2_8)=11　11月
(q28_2_8)=12　12月

【第9次】

(q28_2_9)=　▽以下から選択
(q28_2_9)=01　1月
(q28_2_9)=02　2月
(q28_2_9)=03　3月
(q28_2_9)=04　4月
(q28_2_9)=05　5月
(q28_2_9)=06　6月
(q28_2_9)=07　7月
(q28_2_9)=08　8月
(q28_2_9)=09　9月
(q28_2_9)=10　10月
(q28_2_9)=11　11月
(q28_2_9)=12　12月

【第10次】

(q28_2_10)= ▽以下から選択
(q28_2_10)=01 1月
(q28_2_10)=02 2月
(q28_2_10)=03 3月
(q28_2_10)=04 4月
(q28_2_10)=05 5月
(q28_2_10)=06 6月
(q28_2_10)=07 7月
(q28_2_10)=08 8月
(q28_2_10)=09 9月
(q28_2_10)=10 10月
(q28_2_10)=11 11月
(q28_2_10)=12 12月

【第11次】

(q28_2_11)= ▽以下から選択
(q28_2_11)=01 1月
(q28_2_11)=02 2月
(q28_2_11)=03 3月
(q28_2_11)=04 4月
(q28_2_11)=05 5月
(q28_2_11)=06 6月
(q28_2_11)=07 7月
(q28_2_11)=08 8月
(q28_2_11)=09 9月
(q28_2_11)=10 10月
(q28_2_11)=11 11月
(q28_2_11)=12 12月

【第12次】

(q28_2_12)=　▽以下から選択
(q28_2_12)=01　1月
(q28_2_12)=02　2月
(q28_2_12)=03　3月
(q28_2_12)=04　4月
(q28_2_12)=05　5月
(q28_2_12)=06　6月
(q28_2_12)=07　7月
(q28_2_12)=08　8月
(q28_2_12)=09　9月
(q28_2_12)=10　10月
(q28_2_12)=11　11月
(q28_2_12)=12　12月

【第13次】

(q28_2_13)=　▽以下から選択
(q28_2_13)=01　1月
(q28_2_13)=02　2月
(q28_2_13)=03　3月
(q28_2_13)=04　4月
(q28_2_13)=05　5月
(q28_2_13)=06　6月
(q28_2_13)=07　7月
(q28_2_13)=08　8月
(q28_2_13)=09　9月
(q28_2_13)=10　10月
(q28_2_13)=11　11月

(q28_2_13)=12　12月

【第14次】

(q28_2_14)=　▽以下から選択
(q28_2_14)=01　1月
(q28_2_14)=02　2月
(q28_2_14)=03　3月
(q28_2_14)=04　4月
(q28_2_14)=05　5月
(q28_2_14)=06　6月
(q28_2_14)=07　7月
(q28_2_14)=08　8月
(q28_2_14)=09　9月
(q28_2_14)=10　10月
(q28_2_14)=11　11月
(q28_2_14)=12　12月

【第15次】

(q28_2_15)=　▽以下から選択
(q28_2_15)=01　1月
(q28_2_15)=02　2月
(q28_2_15)=03　3月
(q28_2_15)=04　4月
(q28_2_15)=05　5月
(q28_2_15)=06　6月
(q28_2_15)=07　7月
(q28_2_15)=08　8月
(q28_2_15)=09　9月
(q28_2_15)=10　10月

(q28_2_15)=11 11月

(q28_2_15)=12 12月

【第16次】

(q28_2_16)= ▽以下から選択

(q28_2_16)=01 1月

(q28_2_16)=02 2月

(q28_2_16)=03 3月

(q28_2_16)=04 4月

(q28_2_16)=05 5月

(q28_2_16)=06 6月

(q28_2_16)=07 7月

(q28_2_16)=08 8月

(q28_2_16)=09 9月

(q28_2_16)=10 10月

(q28_2_16)=11 11月

(q28_2_16)=12 12月

このページに入力したものを全てクリアする

戻る 次へ

（ファイル名 q28_2.htm）

- 回答内容により質問番号が飛ぶことがありますが、気にせずにご回答ください。
- 回線の混雑などが原因で反応が遅くなることがあります。ご了承ください。
- ブラウザの戻るボタンで戻った場合、回答が正しく受け付けられない場合があります。その場合、再度アンケートを最初からやり直して下さい。

（調査NO. 12-811-0045）

NIKKEI-R

(pid)

「世界に貢献する環境経済の政策研究」における生物多様性とコミュニティーとの関わりの調査

Q28_3

当選された場合は「当選」に、落選された場合は「落選」を選択してください。 《それぞれひとつずつ》

	当選	落選
第1次	(q28_3_01)=1	(q28_3_01)=2
第2次	(q28_3_02)=1	(q28_3_02)=2
第3次	(q28_3_03)=1	(q28_3_03)=2
第4次	(q28_3_04)=1	(q28_3_04)=2
第5次	(q28_3_05)=1	(q28_3_05)=2
第6次	(q28_3_06)=1	(q28_3_06)=2
第7次	(q28_3_07)=1	(q28_3_07)=2
第8次	(q28_3_08)=1	(q28_3_08)=2
第9次	(q28_3_09)=1	(q28_3_09)=2
第10次	(q28_3_10)=1	(q28_3_10)=2
第11次	(q28_3_11)=1	(q28_3_11)=2
第12次	(q28_3_12)=1	(q28_3_12)=2
第13次	(q28_3_13)=1	(q28_3_13)=2
第14次	(q28_3_14)=1	(q28_3_14)=2
第15次	(q28_3_15)=1	(q28_3_15)=2
第16次	(q28_3_16)=1	(q28_3_16)=2

このページに入力したものを全てクリアする

戻る　次へ

（ファイル名 q28_3.htm）

- 回答内容により質問番号が飛ぶことがありますが、気にせずにご回答ください。
- 回線の混雑などが原因で反応が遅くなることがあります。ご了承ください。
- ブラウザの戻るボタンで戻った場合、回答が正しく受け付けられない場合があります。その場合、再度アンケートを最初からやり直して下さい。

（調査NO. 12-811-0045）

(pid)

「世界に貢献する環境経済の政策研究」における生物多様性とコミュニティーとの関わりの調査

Q28_4

当選された場合、入居された場合は「入居」に、辞退された場合は「辞退」を選択してください。 《それぞれひとつずつ》

	入居	辞退
第1次	(q28_4_01)=1	(q28_4_01)=2
第2次	(q28_4_02)=1	(q28_4_02)=2
第3次	(q28_4_03)=1	(q28_4_03)=2
第4次	(q28_4_04)=1	(q28_4_04)=2
第5次	(q28_4_05)=1	(q28_4_05)=2
第6次	(q28_4_06)=1	(q28_4_06)=2
第7次	(q28_4_07)=1	(q28_4_07)=2
第8次	(q28_4_08)=1	(q28_4_08)=2
第9次	(q28_4_09)=1	(q28_4_09)=2
第10次	(q28_4_10)=1	(q28_4_10)=2
第11次	(q28_4_11)=1	(q28_4_11)=2
第12次	(q28_4_12)=1	(q28_4_12)=2
第13次	(q28_4_13)=1	(q28_4_13)=2

第14次	(q28_4_14)=1	(q28_4_14)=2
第15次	(q28_4_15)=1	(q28_4_15)=2
第16次	(q28_4_16)=1	(q28_4_16)=2

このページに入力したものを全てクリアする

戻る　次へ

（ファイル名 q28_4.htm）

- 回答内容により質問番号が飛ぶことがありますが、気にせずにご回答ください。
- 回線の混雑などが原因で反応が遅くなることがあります。ご了承ください。
- ブラウザの戻るボタンで戻った場合、回答が正しく受け付けられない場合があります。その場合、再度アンケートを最初からやり直して下さい。

（調査NO. 12-811-0045）

NIKKEI-R

(pid)

「世界に貢献する環境経済の政策研究」における生物多様性とコミュニティーとの関わりの調査

Q29

現在あなたのご家族の高齢者の構成についてお伺いします。あなたのお宅に介護の必要な方はおられますか。　《ひとつだけ》

(q29)=1 はい
(q29)=2 いいえ

Q30

震災以前、あなたはパソコンを持っていましたか。　《ひとつだけ》

(q30)=1 はい
(q30)=2 いいえ

Q31

震災以前、あなたはインターネットを日にどの程度利用していましたか。　《ひとつだけ》

(q31)=1 回数は分からないが、パソコン又は携帯電話でEメールのチェックだけをしていた

(q31)=2 Eメールのチェックと、ポータルサイト（たとえばYahoo! JapanやMSN Japan、Infoseek、Biglobeなど）でのニュースのチェックをしていた

(q31)=3 Eメールのチェックとポータルサイトでのニュースのチェック以外に、ネットサーフィンなどを行って情報収集をしていた

(q31)=4 インターネットは使っていなかった

Q32

あなたは今後以前の居住地に戻ることを計画していますか。 （ひとつだけ）

(q32)=1 はい

(q32)=2 いいえ

このページに入力したものを全てクリアする

戻る 次へ

（ファイル名 q29.htm）

- 回答内容により質問番号が飛ぶことがありますが、気にせずにご回答ください。
- 回線の混雑などが原因で反応が遅くなることがあります。ご了承ください。
- ブラウザの戻るボタンで戻った場合、回答が正しく受け付けられない場合があります。その場合、再度アンケートを最初からやり直して下さい。

（調査NO. 12-811-0045）

NIKKEI-R

(pid)

「世界に貢献する環境経済の政策研究」における生物多様性とコミュニティーとの関わりの調査

【 Q32. で「はい」とお答えの方へ 】

Q33

考えられる理由を上位３つまで選んでください。 （上位から順番にお答えください）

自分が生まれ育った土地であり、戻ることが可能である
自分と関わりが深い人たちも、そこに戻ることが分かっている
自分の資産や生業の基盤がある
新しい土地での生活はなじめそうにない
復興を自分の手で成功させたい
その他

１番目： (q33_1)= ▽以下から選択
(q33_1)=1 自分が生まれ育った土地であり、戻ることが可能である

(q33_1)=2 自分と関わりが深い人たちも、そこに戻ることが分かっている

(q33_1)=3 自分の資産や生業の基盤がある

(q33_1)=4 新しい土地での生活はなじめそうにない

(q33_1)=5 復興を自分の手で成功させたい

(q33_1)=6 その他

２番目：

(q33_2)= ▽以下から選択
(q33_2)=1 自分が生まれ育った土地であり、戻ることが可能である

(q33_2)=2 自分と関わりが深い人たちも、そこに戻ることが分かっている

(q33_2)=3 自分の資産や生業の基盤がある

(q33_2)=4 新しい土地での生活はなじめそうにない

(q33_2)=5 復興を自分の手で成功させたい

(q33_2)=6 その他

３番目：

(q33_3)= ▽以下から選択
(q33_3)=1 自分が生まれ育った土地であり、戻ることが可能である

(q33_3)=2 自分と関わりが深い人たちも、そこに戻ることが分かっている

(q33_3)=3 自分の資産や生業の基盤がある

(q33_3)=4 新しい土地での生活はなじめそうにない

(q33_3)=5 復興を自分の手で成功させたい

(q33_3)=6 その他

▶ このページに入力したものを全てクリアする

◀ 戻る　次へ ▶

（ファイル名 q33.htm）

- 回答内容により質問番号が飛ぶことがありますが、気にせずにご回答ください。
- 回線の混雑などが原因で反応が遅くなることがあります。ご了承ください。
- ブラウザの戻るボタンで戻った場合、回答が正しく受け付けられない場合があります。その場合、再度アンケートを最初からやり直して下さい。

（調査NO.12-811-0045）

NIKKEI-R

(pid)

「世界に貢献する環境経済の政策研究」における生物多様性とコミュニティーとの関わりの調査

Q33SQ1

Q33で「その他」とご回答の方は具体的にご記入ください。

（半角カタカナは使用しないでください）

(q33sq1x)

このページに入力したものを全てクリアする

戻る 次へ

（ファイル名 q33sq1.htm）

- 回答内容により質問番号が飛ぶことがありますが、気にせずにご回答ください。
- 回線の混雑などが原因で反応が遅くなることがあります。ご了承ください。
- ブラウザの戻るボタンで戻った場合、回答が正しく受け付けられない場合があります。その場合、再度アンケートを最初からやり直して下さい。

（調査NO. 12-811-0045）

(pid)

「世界に貢献する環境経済の政策研究」における生物多様性とコミュニティーとの関わりの調査

【 Q32. で「いいえ」とお答えの方へ 】

Q34

考えられる理由を上位３つまで選んでください。

（上位から順番にお答えください）

自分が生まれ育った土地だが、戻ることが不可能である
自分と関わりが深い人たちが、そこに戻ることが不明である
自分の資産や生業の基盤がない
震災の記憶から身を離して、新しい土地での生活を始めたい
その他

１番目：

(q34_1)= ▽以下から選択
(q34_1)=1 自分が生まれ育った土地だが、戻ることが不可能である
(q34_1)=2 自分と関わりが深い人たちが、そこに戻ることが不明である
(q34_1)=3 自分の資産や生業の基盤がない
(q34_1)=4 震災の記憶から身を離して、新しい土地での生活を始めたい
(q34_1)=5 その他

２番目：

(q34_2)= ▽以下から選択
(q34_2)=1 自分が生まれ育った土地だが、戻ることが不可能である

(q34_2)=2 自分と関わりが深い人たちが、そこに戻ることが不明である

(q34_2)=3 自分の資産や生業の基盤がない

(q34_2)=4 震災の記憶から身を離して、新しい土地での生活を始めたい

(q34_2)=5 その他

３番目：

(q34_3)= ▽以下から選択
(q34_3)=1 自分が生まれ育った土地だが、戻ることが不可能である

(q34_3)=2 自分と関わりが深い人たちが、そこに戻ることが不明である

(q34_3)=3 自分の資産や生業の基盤がない

(q34_3)=4 震災の記憶から身を離して、新しい土地での生活を始めたい

(q34_3)=5 その他

このページに入力したものを全てクリアする

戻る 次へ

（ファイル名 q34.htm）

- 回答内容により質問番号が飛ぶことがありますが、気にせずにご回答ください。
- 回線の混雑などが原因で反応が遅くなることがあります。ご了承ください。
- ブラウザの戻るボタンで戻った場合、回答が正しく受け付けられない場合があります。その場合、再度アンケートを最初からやり直して下さい。

（調査NO. 12-811-0045）

NIKKEI-R

(pid)

「世界に貢献する環境経済の政策研究」における生物多様性とコミュニティーとの関わりの調査

Q34SQ1

Q34で「その他」とご回答の方は具体的にご記入ください。

（半角カタカナは使用しないでください）

(q34sq1x)

このページに入力したものを全てクリアする

戻る 次へ

（ファイル名 q34sq1.htm）

- 回答内容により質問番号が飛ぶことがありますが、気にせずにご回答ください。
- 回線の混雑などが原因で反応が遅くなることがあります。ご了承ください。
- ブラウザの戻るボタンで戻った場合、回答が正しく受け付けられない場合があります。その場合、再度アンケートを最初からやり直して下さい。

（調査NO. 12-811-0045）

(pid)

「世界に貢献する環境経済の政策研究」における生物多様性とコミュニティーとの関わりの調査

Q35

今回の震災以降、あなたの自然に対する考え方はどのように変化しましたか。 （いくつでも）

(q35_1)=1 自然に対する恐怖を抱き、堤防や耐震建築などで身を守る必要性を感じた
(q35_2)=1 自然への拒否感が強くなり、できるだけ距離を置きたいと感じた
(q35_3)=1 自然の脅威を知り、災害に対して柔軟な対応ができるコミュニティの重要性を認識した
(q35_4)=1 自然は強く美しいものであり、大切に保護すべきものだと感じた
(q35_5)=1 自然は各地の特性が強いものであり、改めて地元の自然に愛着を感じた
(q35_6)=1 その他 （具体的に： (q35_6x) ）

このページに入力したものを全てクリアする

戻る　次へ

（ファイル名 q35.htm）

- 回答内容により質問番号が飛ぶことがありますが、気にせずにご回答ください。
- 回線の混雑などが原因で反応が遅くなることがあります。ご了承ください。
- ブラウザの戻るボタンで戻った場合、回答が正しく受け付けられない場合があります。その場合、再度アンケートを最初からやり直して下さい。

（調査NO. 12-811-0045）

(pid)

「世界に貢献する環境経済の政策研究」における生物多様性とコミュニティーとの関わりの調査

Q36

あなたが復興計画において重要だと考えるものを上位3つまで選んでください。 （上位から順番にお答えください）

地域経済の成長

地域における雇用促進
地域インフラの整備
失業保険等の社会保障の充実
地域的連携の強化
地域医療・教育の拡充
気候変動対策の推進
地域生態系の保全
地域におけるリサイクルの促進
その他

１番目に重要：

(q36_1)＝　▽以下から選択
(q36_1)=01　地域経済の成長
(q36_1)=02　地域における雇用促進
(q36_1)=03　地域インフラの整備
(q36_1)=04　失業保険等の社会保障の充実
(q36_1)=05　地域的連携の強化
(q36_1)=06　地域医療・教育の拡充
(q36_1)=07　気候変動対策の推進
(q36_1)=08　地域生態系の保全
(q36_1)=09　地域におけるリサイクルの促進
(q36_1)=10　その他

２番目に重要：

(q36_2)＝　▽以下から選択
(q36_2)=01　地域経済の成長
(q36_2)=02　地域における雇用促進
(q36_2)=03　地域インフラの整備
(q36_2)=04　失業保険等の社会保障の充実
(q36_2)=05　地域的連携の強化
(q36_2)=06　地域医療・教育の拡充
(q36_2)=07　気候変動対策の推進
(q36_2)=08　地域生態系の保全
(q36_2)=09　地域におけるリサイクルの促進
(q36_2)=10　その他

３番目に重要：

(q36_3)＝　▽以下から選択
(q36_3)=01　地域経済の成長
(q36_3)=02　地域における雇用促進
(q36_3)=03　地域インフラの整備

(q36_3)=04 失業保険等の社会保障の充実

(q36_3)=05 地域的連携の強化

(q36_3)=06 地域医療・教育の拡充

(q36_3)=07 気候変動対策の推進

(q36_3)=08 地域生態系の保全

(q36_3)=09 地域におけるリサイクルの促進

(q36_3)=10 その他

このページに入力したものを全てクリアする

戻る 次へ

（ファイル名 q36.htm）

- 回答内容により質問番号が飛ぶことがありますが、気にせずにご回答ください。
- 回線の混雑などが原因で反応が遅くなることがあります。ご了承ください。
- ブラウザの戻るボタンで戻った場合、回答が正しく受け付けられない場合があります。その場合、再度アンケートを最初からやり直して下さい。

（調査NO. 12-811-0045）

NIKKEI-R

(pid)

「世界に貢献する環境経済の政策研究」における生物多様性とコミュニティーとの関わりの調査

Q36SQ1

Q36で「その他」とご回答の方は具体的にご記入ください。

（半角カタカナは使用しないでください）

(q36sq1x)

このページに入力したものを全てクリアする

戻る 次へ

（ファイル名 q36sq1.htm）

- 回答内容により質問番号が飛ぶことがありますが、気にせずにご回答ください。
- 回線の混雑などが原因で反応が遅くなることがあります。ご了承ください。
- ブラウザの戻るボタンで戻った場合、回答が正しく受け付けられない場合があります。その場合、再度アンケートを最初からやり直して下さい。

(調査NO. 12-811-0045)

(pid)

「世界に貢献する環境経済の政策研究」における生物多様性とコミュニティーとの関わりの調査

Q37

今後の災害対策において、下記のそれぞれの対策をどのくらい重要だと考えますか。それぞれ5段階で選んで下さい。 《それぞれひとつずつ》

	重要	←	·	→	重要でない
	5	4	3	2	1
人工物による防御<br>例：堤防	(q37_1)=5	(q37_1)=4	(q37_1)=3	(q37_1)=2	(q37_1)=1
自然を活かした減災<br>例：防潮林	(q37_2)=5	(q37_2)=4	(q37_2)=3	(q37_2)=2	(q37_2)=1
土地利用の改善<br>例：高台移転	(q37_3)=5	(q37_3)=4	(q37_3)=3	(q37_3)=2	(q37_3)=1
地域内の連携強化<br>例：防災訓練	(q37_4)=5	(q37_4)=4	(q37_4)=3	(q37_4)=2	(q37_4)=1
地域間の連携強化<br>例：情報共有	(q37_5)=5	(q37_5)=4	(q37_5)=3	(q37_5)=2	(q37_5)=1

このページに入力したものを全てクリアする

戻る 次へ

(ファイル名 q37.htm)

- 回答内容により質問番号が飛ぶことがありますが、気にせずにご回答ください。
- 回線の混雑などが原因で反応が遅くなることがあります。ご了承ください。
- ブラウザの戻るボタンで戻った場合、回答が正しく受け付けられない場合があります。その場合、再度アンケートを最初からやり直して下さい。

(調査NO. 12-811-0045)

(pid)

「世界に貢献する環境経済の政策研究」における生物多様性とコミュニティーとの関わりの調査

Q38

平成23年度第三次補正予算案では、被災した漁港の復旧事業など漁業の復興に対し、およそ6,300億円の予算を計上しています。あなたは、津波で被災した漁港について、次の対策をどのように考えますか。最も望ましいと思うものを下記選択肢より１つ選んでください。 （ひとつだけ）

対策A：被災した漁港・水産加工施設を復旧し、従来の漁業の再生を目指す<br>対策B：漁港には戻さず、海洋沿岸保護区を設立して観光や研究を促進する

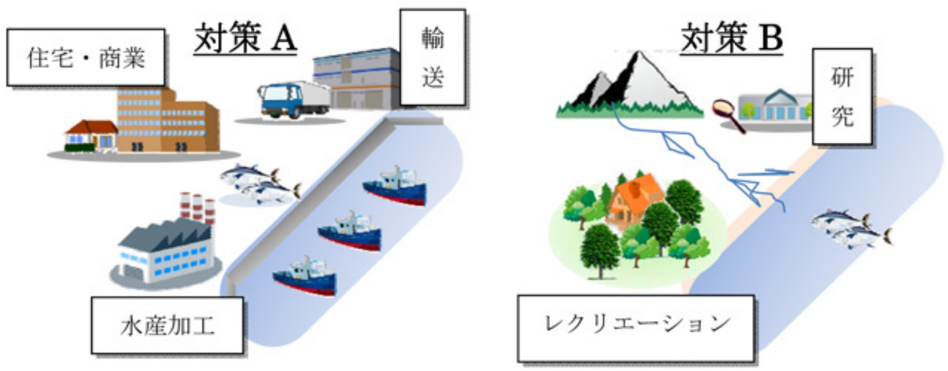

(q38)=1 この予算をすべて対策Aに支出すべき

(q38)=2 この予算の75%を対策Aに、25%を対策Bに支出すべき

(q38)=3 この予算の50%を対策Aに、50%を対策Bに支出すべき

(q38)=4 この予算の25%を対策Aに、75%を対策Bに支出すべき

(q38)=5 この予算をすべて対策Bに支出すべき

(q38)=6 その他 （具体的に：(q38_6x) ）

Q39

平成23年度第三次補正予算案では、被災した農地の復旧および区画整理事業に対し、2,080億円の予算を計上しています。あなたは、被災した農地について、次の対策をどのように考えますか。最も望ましいと思うものを下記選択肢より１つ選んでください。 （ひとつだけ）

対策A：農地全域において用水路・排水路設置作業や除塩対策を含む復旧事業を行うとともに、大区画化による営農を促進する<br>対策B：沿岸線に近い農地においては、地元の樹種を活用した防潮林の育成を進め、生態系による水質浄化・災害緩和機能を強化する

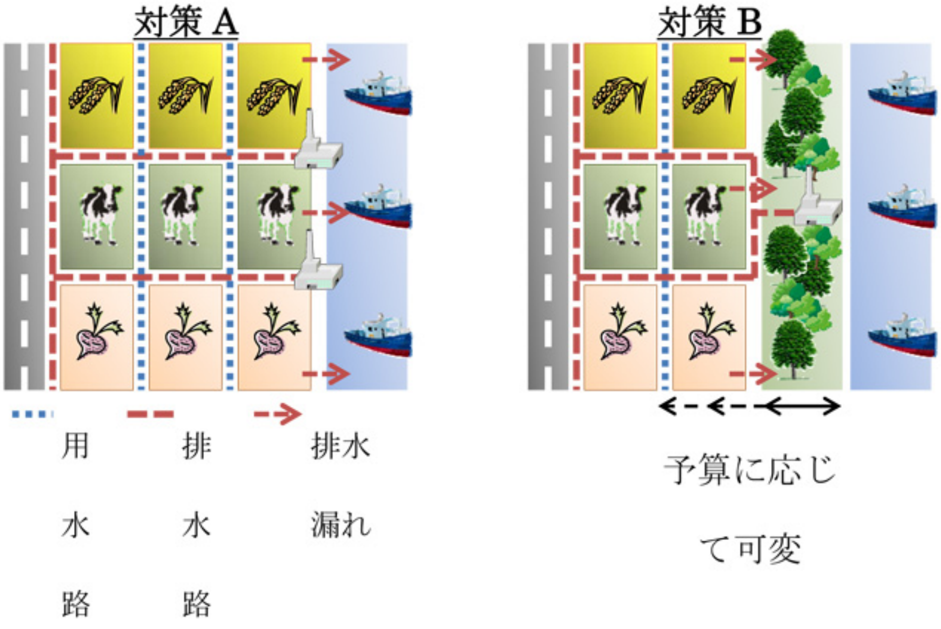

(q39)=1 この予算をすべて対策Aに支出すべき
(q39)=2 この予算の90%を対策Aに、10%を対策Bに支出すべき
(q39)=3 この予算の80%を対策Aに、20%を対策Bに支出すべき
(q39)=4 この予算の70%を対策Aに、30%を対策Bに支出すべき
(q39)=5 この予算の60%を対策Aに、40%を対策Bに支出すべき
(q39)=6 この予算の50%を対策Aに、50%を対策Bに支出すべき
(q39)=7 その他 （具体的に：(q39_7x) ）

戻る　送信　このページに入力したものを全てクリアする

（ファイル名 q38.htm）

- 回答内容により質問番号が飛ぶことがありますが、気にせずにご回答ください。
- 回線の混雑などが原因で反応が遅くなることがあります。ご了承ください。
- ブラウザの戻るボタンで戻った場合、回答が正しく受け付けられない場合があります。その場合、再度アンケートを最初からやり直して下さい。

（調査NO. 12-811-0045）

NIKKEI-R
